# Clinical laboratory diagnostic stewardship as boundary infrastructure: a multi-stakeholder qualitative study of digital public health governance in two Chinese regional referral hospitals

**DOI:** 10.3389/fpubh.2026.1894293

**Published:** 2026-09-28

**Authors:** Long Yao, Zhuojun Deng, Fuhua Luo, Lei He

**Affiliations:** 1 Department of Laboratory Medicine, The First Affiliated Hospital of Shaoyang University, Shaoyang, Hunan, China; 2 Department of Laboratory Medicine, The Second Affiliated Hospital of Shaoyang University, Shaoyang, Hunan, China; 3 Department of Radiology, The First Affiliated Hospital of Shaoyang University, Shaoyang, Hunan, China

**Keywords:** accountable handoff, boundary infrastructure, clinical laboratory medicine, diagnostic stewardship, digital public health, ISO 15189, laboratory information system, qualitative research

## Abstract

**Background:**

Digital public health within population-serving hospitals depends on governance that connects diagnostic information to accountable action. This study examined how digital systems, interpretive expertise, professional authority, and communication practices jointly organized clinical laboratory diagnostic stewardship.

**Methods:**

An interpretive, multi-stakeholder, contrasting-case qualitative study was conducted from May 2025 to January 2026 in two purposefully selected tertiary Grade-A hospitals in Shaoyang, China. A critical realist position combined attention to organizational conditions with a fallibilist, interpretive epistemology. Forty-six stakeholders completed semi-structured interviews; 36 contextual workflow documents supported process mapping. Chinese transcripts were analyzed abductively using reflexive thematic analysis, role- and site-comparison matrices, and discrepant-case analysis. SRQR guided overall reporting and COREQ supplemented interview-specific disclosure. Anatomical pathology was excluded.

**Results:**

Four interdependent functions were developed: epistemic qualification; jurisdictional authorization; accountable handoff; and recursive institutionalization and learning. Participants associated Hospital A’s accredited, more formalized configuration with traceability and reproducibility, but also with generic comments, alert burden, and procedural closure. Hospital B’s more relationship-dependent configuration was associated with contextual responsiveness and access to known experts, but also with person-dependence, weaker after-hours coverage, and limited auditability. Accounts of nurse-mediated relay, ordering friction, contextual interpretation, and rule ownership supported a cross-cutting explanation of how formal arrangements and accessible expertise complemented one another. Proposed evaluation indicators were not measured outcomes.

**Conclusion:**

Diagnostic stewardship is interpreted as boundary infrastructure connecting evidentiary limits, advisory authority, action ownership, service coverage, and rule learning. The study provides context-bounded explanatory propositions and a separate prospective evaluation agenda, not estimates of effectiveness or hospital performance.

## 1 Introduction

Laboratory testing creates clinical and public-health value only when an appropriate test is selected, a suitable specimen is obtained, analytical quality is secured, the result is interpreted in context, and a responsible actor completes the next action. Overuse can expose patients and services to unnecessary phlebotomy, false-positive cascades, avoidable cost, and attention diverted from higher-value care; underuse can delay diagnosis, treatment, or prevention. Many consequential failures arise before analysis or after result release rather than at the analytical bench (1–7).

Diagnostic stewardship addresses this total testing pathway. The study defines diagnostic stewardship as coordinated work that improves the appropriateness, reliability, interpretation, communication, and follow-up of diagnostic testing (8–11). Test-utilization management is a narrower component concerned with test selection, repeat testing, and alignment with clinical indication (12–14). Value-based laboratory medicine asks whether laboratory activity contributes to better decisions, outcomes, resource use, and patient experience rather than merely producing more or faster results (15, 16). These perspectives converge on a central problem: technically valid data do not create value unless uncertainty, authority, responsibility, and follow-up are organized across the entire pathway.

Diagnostic stewardship is consequently a distributed governance practice rather than a discrete activity owned by one profession. The appropriateness of an order may depend on clinical indication and prior results; specimen integrity depends on collection and transport; interpretation depends on analytical limitations and clinical context; and follow-up depends on a receiver who has both access and authority. Interventions that optimize only one node can displace rather than resolve risk. For example, a stricter electronic restriction may reduce duplicate orders while delaying a justified repeat, and faster notification may increase recorded compliance while leaving the next decision unowned. A governance model must therefore connect the informational, professional, temporal, and organizational conditions of the pathway.

Digital public health concerns the transformation of digital systems to support public-health functions, service organization, population needs, and accountable decision-making rather than the digitization of records alone (17–19). In regional referral hospitals that serve urban and surrounding-county populations, laboratory information system and electronic medical record integration, electronic ordering, decision support, acknowledgment functions, dashboards, and interoperability distribute diagnostic resources and scarce interpretive expertise across departments, shifts, and large catchments. Sociotechnical and scale-up frameworks show that technology remains inseparable from users, workflow, organizational rules, and wider-system conditions and that adoption does not guarantee sustained, safe, or equitable use (20, 21). The relevant unit of analysis is therefore institution-level governance of diagnostic information, expertise, responsibility, coverage, and workload, not the isolated performance of one alert or software module.

Digital artifacts are not self-executing. A duplicate-order prompt requires an evidence base, a defined clinical population, an understandable rationale, justified override, monitoring for missed necessary testing, and a process for revision. A critical-result system may document transmission or acknowledgment without establishing whether an authorized actor understood the result and completed a clinical response. Report comments can standardize interpretive support while becoming generic or noisy; telephone consultation can provide context-sensitive advice while remaining person-dependent and difficult to audit. These tensions explain why electronic deployment can improve traceability yet still produce alert fatigue, unsafe blocking, unequal after-hours access, or the appearance of closure without owned action (22–24).

The work is also distributed across professional jurisdictions. Laboratory personnel know analytical limitations, assay interference, specimen quality, and appropriate confirmation. Clinicians integrate these inputs with examination, imaging, treatment response, and patient preference. Nurses and specimen-process coordinators often hold the communication chain together; information-system personnel translate clinical intentions into software; quality and medical-administration staff assign ownership and review performance. International qualitative studies show that the visibility, authority, and accessibility of laboratory expertise vary across health systems (25–27). The potentially transferable problem is therefore functional: what must occur for diagnostic information to become usable and owned, even when occupational titles and communication pathways differ.

Existing diagnostic-stewardship research has generated important guidance on test selection, antimicrobial diagnostics, electronic decision support, and utilization management (8–16, 22–24). Less attention has been paid to the organizational mechanisms that connect an uncertain result to an authorized interpretation, move responsibility beyond the first receipt, and keep a digital rule clinically owned after implementation. Qualitative studies of laboratory-clinician interfaces reveal communication and recognition problems (25–27), but they do not yet provide a unified account linking meaning, jurisdiction, handoff, recursive learning, and measurable failure conditions. That gap matters for digital public health because large-scale data flow can amplify both reliable coordination and systematic responsibility loss.

Boundary infrastructure provides the primary conceptual framework. Infrastructure scholarship explains how artifacts, classifications, roles, routines, standards, and technologies become durable when embedded across communities of practice and how infrastructure becomes visible at breakdown (28–32). Boundary-work scholarship explains professional jurisdiction (33, 34), while invisible-work research directs attention to labor absent from reports and dashboards (28). The term boundary infrastructure predates the study and has been applied to health-information integration, including laboratory-primary-care interfaces (31, 35). The claimed novelty is therefore functional and empirical rather than terminological. Sittig and Singh offer a system-wide sociotechnical model; NASSS explains nonadoption, abandonment, scale, spread, and sustainability; and ISO 15189, its identical adoption in CNAS-CL02:2023, and Chinese clinical-laboratory regulation establish normative expectations for competence, authority, information management, risk, and continual improvement (20, 21, 36–38). What remains insufficiently specified is the diagnostic-stewardship work required when a request, specimen, or result crosses professional and digital boundaries, the failure conditions that reveal each function, and the prospective indicators that could test them.

China’s institutional setting is consequential but not universal. Hospitals are classified administratively by tier and grade; tertiary is the highest service tier and Grade A the highest grade within that tier. Government review associates this designation with complex and emergency care, specialist capacity, teaching, regional support, quality management, and data governance, but it is not directly equivalent to a single hospital category elsewhere and does not imply identical staffing, accreditation, or digital maturity (39, 40). Diagnostic error and overuse remain system-level concerns (41–45), while continuing health-system reform and diffusion of organizational innovation shape regional service capacity (46–48). Prefecture-level tertiary hospitals are therefore analytically important sites for examining how population-serving institutions govern high-volume diagnostic information without assuming that their role structures should be generalized unchanged.

The study addressed the following primary question: how do hospital stakeholders convert a clinical laboratory request, specimen, or result requiring cross-boundary interpretation or action into an accountable next laboratory or clinical action without blurring diagnostic authority? Subsidiary questions examined how the functions were developed from participant accounts and document-supported cross-site comparison; how workforce, accreditation, and quality-governance conditions shaped them; which expressions were specific to the two Chinese hospitals; why formal digital arrangements and relationship-based consultation remained incomplete when used alone; and how the resulting model could be adapted and tested in other systems. The study contributes a transparent chain from reported events to four functions, paired failure conditions, and prospective indicator families while maintaining a clear boundary between provisional qualitative explanations and future outcome evaluation.

## 2 Materials and methods

### 2.1 Study design, scope, and analytic orientation

The study used an interpretive, multi-stakeholder, contrasting-case qualitative design, with critical realism as the philosophical position for its explanatory account, combining semi-structured interviews with contextual document review. Its purpose was to explain the work, tensions, breakdowns, and governance conditions through which laboratory requests, specimens, and results became, or failed to become, actionable in routine care. The empirical scope comprised clinical chemistry, hematology and coagulation, immunology and serology, clinical microbiology, molecular diagnostics, order and prior-result review, specimen reception and preanalytics, report review, critical-result communication, and laboratory quality management. Anatomical pathology, histopathology, and cytology were organizationally separate and were not sampled or analyzed.

Contrasting-case comparison developed and bounded explanation rather than estimating a site effect. Similar stewardship problems were examined under different bundles of digital formalization, staffing, accreditation, leadership, and relationship-based access. Cross-role accounts and contextual process evidence informed site interpretations, with exceptions actively examined. Recurrence strengthened the basis for examining a pattern but was not a vote establishing its truth; accounts confined to one role could expose an important limitation or responsibility gap. Similarities also mattered because they suggested functions relevant across configurations. Hospital A and Hospital B were therefore not treated as proxies for technology and relationships.

The study constitutes a formative contribution to intervention development within a staged complex-intervention evaluation pathway (49). A mechanism proposition denotes an inferred process connecting organizational conditions with reported patterns of interpretation, responsibility, or action, not an estimated causal effect. The report distinguishes participant-reported events and meanings, cross-case explanatory interpretation, and prospective propositions or indicators requiring validation. The first two levels comprise the qualitative findings. The third is a subsequent design translation, presented in the Discussion (Section 4.7) rather than as a quantitative component of the study. These reporting levels are not interchangeable with direct observations of causal mechanisms.

Reflexive thematic analysis was selected because the study sought patterned meanings across professional roles and sites rather than a coding-reliability product (50–52). Analysis was abductive: event-centered codes were generated from participant accounts and iteratively interpreted through boundary infrastructure, and the four functions were not predetermined coding categories. Diagnostic stewardship and the total testing process oriented empirical inquiry. Boundary infrastructure supplied the principal explanatory vocabulary; boundary work and invisible work supported interpretation of authority and hidden labor. Sittig-Singh and NASSS sensitized analysis to sociotechnical configuration and scale, whereas ISO 15189 informed post-theme interpretation of competence, responsibility, information governance, risk, and continual improvement. These conceptual and normative resources were not substitutes for an epistemological position or templates into which accounts had to fit.

### 2.2 Philosophical assumptions and epistemological position

The explanatory interpretation is situated within critical realism, combining a realist ontology with an interpretive, fallibilist epistemology (53). Staffing, competency authorization, digital rules, and escalation routes can enable or constrain action beyond any single participant’s account. Knowledge of their operation is nevertheless situated and mediated by professional experience, interview interaction, language, and researchers’ theoretical commitments. Socially constituted authority can have material consequences without either interviews or documents providing an unmediated account of those consequences. This position fits the question of how organizational arrangements shape diagnostic work; it is not selected merely because future indicators can be expressed numerically. Neither laboratory perspectives nor formal records are privileged as the definitive account of practice.

The distinction between experience, events, and generative conditions matters when acknowledgment appears complete but responsibility remains unresolved. A workflow template can establish how acknowledgment is intended to be recorded; it cannot establish that a particular patient received the intended action. An interview illuminates what acknowledgment meant to the speaker and how responsibility was experienced, but does not independently reconstruct the patient’s course. The interpretation considers whether receiver availability, recognized authority, access to clinical context, or escalation arrangements plausibly explain the reported gap. Staffing, workload, professional jurisdiction, and software design remain interacting or rival explanations, not variables with separately established effects (53, 54).

Reflexive thematic analysis is the analytic method, not the philosophical paradigm. Themes are researchers’ theoretically informed constructions of patterned meaning, not entities recovered through agreement between interchangeable coders (50–52, 55). A realist explanatory commitment is compatible with this interpretive work when accounts are treated as situated evidence and explanations remain open to challenge. Abduction redescribes reported problems conceptually; cross-role contrasts, exceptions, and contextual documents constrain the resulting interpretation. The four functions organize that interpretation but are not, by being named, demonstrated causal mechanisms. This philosophical articulation does not introduce a separate realist-evaluation protocol, statistical corroboration, or a retrospective change to data collection. The study remains qualitative; its future measurement agenda is analytically separate. Descriptive counts identify the sample and documentary corpus; they do not measure the prevalence or truth of a theme. The candidate numerators and denominators in the prospective evaluation agenda specify future observations, not quantitative verification of the present interpretations.

This positioning has practical consequences for evidence use. A participant’s interpretation, a reported event, and a proposed generative condition are related but not equivalent. Contrasting perspectives can therefore reveal different access to responsibility or information without one account being dismissed as erroneous. Exceptions are useful because they delimit an explanation: an effective direct call challenges the assumption that electronic acknowledgment is intrinsically necessary, whereas an acknowledgment without owned action challenges the assumption that documentation is sufficient. The explanatory question is what makes a route work in a particular context, not which source can be made to certify a context-free rule.

### 2.3 Complementary use of reporting standards

The Standards for Reporting Qualitative Research (SRQR) provide the overarching reporting framework for the research question, qualitative approach and paradigm, reflexivity, context, sampling, ethics, data generation and processing, analysis, empirical support, interpretation, and limitations (56). The Consolidated Criteria for Reporting Qualitative Research (COREQ) supplement SRQR for the interview component: interviewer characteristics and prior relationships, participant approach and recruitment, setting, interview conduct, recording, field notes, analytic responsibilities, participant feedback, and quotation presentation (57). Thus SRQR governs the report’s overall explanatory and evidentiary architecture, while COREQ supplies interview-specific disclosure. Neither checklist determines study design or epistemology; those choices follow the research question and the rationale in Sections 2.1–2.2. Overlapping items cross-reference the same account rather than implying that two different procedures were performed.

Table 1 provides the item-by-item SRQR reporting map, and Table 2 provides the corresponding COREQ map. Both maps identify each item’s manuscript location, corresponding item(s) in the other standard, and disclosure status. The maps are presented as main-text tables, with the full methodological accounts retained in the corresponding sections. Described procedures, explicit non-use, methodological alternatives, and confidentiality-related adaptations are distinguished. Information power replaces saturation as the sampling-adequacy rationale; collaborative interpretation does not entail inter-coder reliability testing; complete transcripts were not returned; and participant feedback concerned resonance rather than verification of a single correct interpretation. Role labels and excerpt identifiers make quoted evidence traceable within the report while protecting identities. Researcher credentials, information provided to participants, and recruitment contact procedures are described in Sections 2.5–2.6 and mapped to the relevant items. The maps document how the study is reported; they are neither quality scores nor evidence that each checklist prompt prescribes a procedure that must be performed.

**Table 1:** Item-by-item SRQR reporting map and overlap with interview-specific COREQ disclosure.

Item	Reporting topic	Disclosure and scope	Location	COREQ overlap
S1	Title	Reported: qualitative approach, laboratory scope, and two-hospital setting.	Title	None
S2	Abstract	Reported: question, design, analysis, principal interpretations, and claim limits.	Abstract	None
S3	Research problem	Reported: diagnostic-stewardship problem, conceptual rationale, and related empirical research.	Section 1	C9 (theory)
S4	Purpose and question	Reported: primary question, subsidiary questions, and formative contribution.	Section 1	None
S5	Approach and paradigm	Reported: contrasting-case design, critical realist ontology, interpretive/fallibilist epistemology, and reflexive thematic analysis.	Sections 2.1–2.3	C9
S6	Researchers and reflexivity	Described: researcher qualifications and training status, roles, participant briefing, relationships, assumptions, and reflexive challenge.	Sections 2.6, 2.8–2.9	C1-C8
S7	Study context	Reported: hospital scope, accreditation, staffing, digital arrangements, and shared context.	Section 2.4; Table 3	C14-C16
S8	Sampling rationale	Described: purposeful variation, eligibility, individual contact channels, recruitment flow, and information power.	Section 2.5; Table 5	C10-C13, C22
S9	Ethics and protection	Reported: approvals, consent, secure storage, confidentiality, and restricted access.	Sections 2.6, 2.10; statements	No direct item
S10	Data generation	Described: individual approach and briefing, interviews, iteration, field notes, documents, and collection period.	Sections 2.4–2.7	C11, C14-C15, C17-C21
S11	Instruments and technology	Reported: guide domains, four retained pilots, recording, and in-person/online interview modes.	Section 2.6	C17, C19
S12	Participants and materials	Reported: 46 interviewees, stakeholder and functional-role profiles, and 36 contextual documents.	Sections 2.5–2.7; Tables 3–5	C12-C13, C16
S13	Data preparation	Reported: Chinese transcription, secure handling, translation checking; no transcript return.	Sections 2.6, 2.8–2.10	C19, C23, C27
S14	Analytic process	Reported: coding roles, comparisons, abductive interpretation, and prospective specification.	Section 2.8; Table 6	C24-C27
S15	Trustworthiness practices	Reported: reflexive memos, discrepant cases, contextual mapping, resonance testing, and audit trail; no agreement statistic.	Sections 2.8–2.9	C8, C28, C30
S16	Synthesis of findings	Reported: four functions, limiting accounts, and an explicitly cross-cutting synthesis.	Section 3; Table 6; Figure 1	C26, C31-C32
S17	Empirical support	Reported: role-labeled translated extracts E1-E9, paraphrased episodes, and interpretation. Reuse is identified.	Sections 2.9, 3; Table 6	C29-C30
S18	Contribution and transfer	Reported: relation to prior scholarship, local expressions, functional transferability, implementation, and future evaluation.	Sections 4.1–4.8; Table 7	No direct item
S19	Study limitations	Reported: selection and professional-position effects, contextual confounding, evidentiary limits, and confidentiality-related reporting constraints.	Section 4.9	C32 (variation)
S20	Competing interests	Reported: competing-interest declaration and professional/institutional positioning.	Conflict; Section 2.9	C6, C8 (overlap)
S21	Funding and support	Reported: no financial support; institutional facilitation acknowledged.	Funding; Acknowledgments	No direct item

All 21 SRQR items are mapped separately. COREQ overlaps identify complementary interview-specific disclosure, not a second procedure. Locations refer to numbered sections and main-text tables. The map is a reporting guide, not a quality score or an independent verification of study procedures. Item numbering follows O’Brien et al. (56).

**Table 2:** Item-by-item COREQ reporting map, complementary SRQR coverage, and explicit disclosure status.

Item	Reporting topic	Disclosure and scope	Location	SRQR overlap
C1	Interviewers	Reported: Long Yao (LY) and Zhuojun Deng (ZD) conducted the interviews.	Section 2.6	S6
C2	Formal credentials	Reported: LY and ZD held bachelor’s degrees in medical laboratory science. FL and LH were master’s students; LH’s program was medical imaging. Enrollment is not a completed master’s degree.	Section 2.6	S6
C3	Occupational roles	Reported: LY and ZD were laboratory-trained researchers affiliated with hospital laboratory departments. FL contributed document work; LH contributed interpretation and project coordination.	Sections 2.6, 2.8	S6
C4	Gender	Reported: LY was female; ZD was male.	Section 2.6	S6
C5	Training and experience	Reported: LY’s qualitative-interviewing training; ZD’s laboratory background and site familiarity; guide piloting and collaborative analytic discussion.	Sections 2.6, 2.8	S6
C6	Prior relationships	Reported: some professional familiarity; disclosed at consent and considered in logs/memos. No direct supervisory authority.	Sections 2.6, 2.9	S6
C7	Participant knowledge of researchers	Described: pre-consent information about researcher backgrounds, academic interests, study purpose, non-evaluative role, and voluntariness, distinct from prior familiarity.	Section 2.6	S6
C8	Interviewer positioning	Reported: laboratory, site-affiliated, document-focused, and medical-imaging perspectives; training positions and professional authority considered reflexively.	Section 2.9	S6, S15
C9	Methodological orientation	Reported: interpretive contrasting-case design, critical realism, boundary infrastructure, and abductive reflexive thematic analysis.	Sections 2.1–2.3	S3, S5
C10	Selection strategy	Reported: purposeful maximum variation, domain eligibility, leadership/liaison identification, and participant referral.	Section 2.5	S8
C11	Approach to candidates	Described: individual face-to-face, telephone, or private WeChat invitations; one further invitation after non-response; no further approach after refusal; recruitment distinguished from interview mode.	Section 2.5	S8, S10
C12	Sample size	Reported: 46 participants, 24 at Hospital A and 22 at Hospital B.	Section 2.5; Table 5	S8, S12
C13	Non-participation	Reported: 7 declined, 5 did not respond after two invitations; no withdrawal after interview.	Section 2.5; Table 5	S8, S12
C14	Interview setting	Reported: 36 face-to-face interviews in private rooms; 10 secure online interviews.	Section 2.6	S7, S10
C15	Other persons present	Explicit non-presence: no non-participants were present.	Section 2.6	S7, S10
C16	Sample description	Reported: stakeholder groups, functional laboratory roles, qualifications, experience, and site distribution.	Sections 2.4–2.5; Tables 3–5	S7, S12
C17	Guide and piloting	Reported: guide domains, episode/counterfactual prompts, iterative refinement, and four retained pilot interviews.	Section 2.6	S10-S11
C18	Repeat interviews	Explicit non-use: no repeat interviews were conducted.	Section 2.9	S10
C19	Recording	Reported: all interviews audio-recorded with permission and transcribed in Chinese.	Section 2.6	S10-S11, S13
C20	Field notes	Reported: context, non-verbal cues, role dynamics, and emerging analytic questions.	Section 2.6	S10
C21	Interview duration	Reported: 42–96 min; median 64 min.	Section 2.6	S10
C22	Sampling adequacy	Methodological alternative: information power, role/site variation, and later boundary testing; data/code saturation was not the stopping criterion.	Sections 2.3, 2.5	S8
C23	Transcript return	Explicit non-use: complete transcripts were not returned. Findings-summary feedback is a separate activity.	Section 2.9	S13
C24	Coding responsibilities	Reported: LY coded the full set; ZD reviewed a purposive subset; FL and LH had contextual/interpretive roles.	Section 2.8	S14
C25	Code-to-theme organization	Reported: evolving analytic labels, comparison matrices, and their relation to four functions; not a fixed reliability coding frame.	Section 2.8; Table 6	S14
C26	Theme development	Reported: functions developed through abductive interpretation, not predetermined thematic categories.	Sections 2.1, 2.8; Table 6	S14, S16
C27	Software	Reported: NVivo 14 for data management and retrieval.	Section 2.8	S13-S14
C28	Participant feedback	Reported: summary discussed with eight participants as resonance testing, not confirmation of a single correct interpretation.	Section 2.9	S15
C29	Quotation identification	Adapted: role labels plus extract identifiers E1-E9; reused extracts retain labels. Individual and site identifiers suppressed for confidentiality.	Sections 2.6, 2.9–2.10, 3.2–3.6	S17
C30	Data-interpretation links	Reported: quotations, paraphrased episodes, explanations, and future propositions distinguished; attribution and evidentiary limits explicit.	Sections 3.2–3.6, 4.9; Table 6	S15, S17
C31	Major findings	Reported: four named functions and paired failure conditions.	Sections 3.1–3.5; Table 6; Figure 1	S16
C32	Variation and exceptions	Reported: resistant/limiting accounts, successful direct-call exception, and departures from dominant site configurations.	Sections 3.2–3.6	S16, S19

Each of the 32 COREQ items is mapped separately. Descriptive reporting, explicit non-use, methodological alternatives, and confidentiality-related adaptations are different statuses, not scores. C2 distinguishes completed degrees from postgraduate enrollment; C7 concerns information given to participants rather than familiarity alone; C11 concerns recruitment contact rather than interview mode. C29 uses excerpt identifiers, not participant identifiers. The map does not independently verify study procedures. Item numbering follows Tong et al. (57).

### 2.4 Setting, laboratory scope, accreditation, and contrasting-case selection

Data collection was conducted from May 2025 to January 2026 at The First Affiliated Hospital of Shaoyang University and The Second Affiliated Hospital of Shaoyang University, referred to as Hospital A and Hospital B. Both were tertiary Grade-A general hospitals reviewed by provincial health authorities under national standards. For international readers, the designation indicates a population-serving institution expected to manage complex and emergency care, provide broad specialist and teaching functions, support lower-level services, and maintain formal quality and information governance. It should not be read as a direct equivalent of an academic medical center, national referral center, or teaching hospital in another system.

Both hospitals served urban Shaoyang and surrounding counties and operated 24-h central clinical laboratory services. They were located in the same prefecture and affiliated with the same university, creating a shared regional regulatory, professional, educational, and labor-market environment. This common environment strengthened contextual comparability but may also have narrowed the range of institutional variation. Table 3 summarizes hospital classification, catchment, laboratory scope and leadership structure, workload and staffing bands, accreditation, digital arrangements, shared institutional context, and the boundaries of case selection.

**Table 3:** Hospital classification, clinical laboratory context, accreditation, shared institutional environment, and contrasting-case logic.

Characteristic	Hospital A	Hospital B	Interpretive boundary
Classification and service role	Tertiary Grade-A general regional referral hospital; 1,400 approved beds; emergency, inpatient, outpatient, specialist, teaching, and regional support.	Tertiary Grade-A general regional referral hospital; 856 approved beds; the same broad service functions.	Highest Chinese tier/grade; not a direct international equivalent or assurance of identical resources.
Catchment	Urban Shaoyang and surrounding counties; emergency, outpatient, inpatient, and referral pathways.	Same urban and surrounding-county catchment through complementary services.	Within-hospital governance, not evaluation of the complete referral network.
Shared environment	Shaoyang Prefecture; affiliated with Shaoyang University.	Shaoyang Prefecture; affiliated with Shaoyang University.	Shared regulation, education, professional networks, referrals, and labor markets may narrow variation.
Clinical laboratory scope	24-h central laboratory; ordering, preanalytics, chemistry, hematology/coagulation, immunology, microbiology, molecular diagnostics, report review, critical results, and quality management.	24-h central laboratory covering the same main disciplines and functions.	Anatomical pathology, histopathology, and cytology excluded.
Leadership and organization	Leadership; section/supervisory; quality/authorized-review; technical/preanalytical functions.	Same functional levels in a smaller central-laboratory workforce.	Authority interpreted through function and competency authorization, not assumed international title equivalence.
12-month workload and staffing	Approximately 2.5–4.0 million analyte-level results; approximately 35–50 central-laboratory personnel.	Approximately 1.0–2.0 million analyte-level results; approximately 20–30 central-laboratory personnel.	Latest complete 12 months at January 2026 check; rounded context bands, not performance rates.
Accreditation and quality	CNAS/ISO 15189:2022 accreditation for defined scope; first granted July 2024. Internal quality control, external quality assessment, competency authorization, audit, and management review.	No ISO 15189 accreditation during study. Internal quality control, external quality assessment, competency authorization, hospital quality procedures, and local document review.	Accreditation co-varied with scale, staffing, leadership, culture, and digital history; no accreditation-effect estimate.
Dominant configuration	More formalized acknowledgment, duplicate-order prompts, specimen procedures, comments, escalation records, and rule review.	Greater reliance on direct consultation and telephone coordination; fewer stable electronic restrictions and less formalized rule review.	Purposefully contrasting configurations, actively tested against exceptions; neither is a causal treatment.
Digital arrangements	Laboratory/electronic medical record integration; selected prior-result displays and override fields; electronic acknowledgment; basic dashboards.	Laboratory/electronic medical record integration; telephone-led relay; local comment templates; more limited rule maintenance.	Digital maturity alone did not establish clinical ownership or consistent coverage.
Contextual workflow documents	21: 4 procedures; 4 templates; 6 rule specifications/screenshots; 2 consultation-log extracts; 5 quality/training records.	15: 3 procedures; 3 templates; 2 rule specifications/screenshots; 3 consultation-log extracts; 4 quality/training records.	Formal process mapping; no patient-level data, performance rates, or accreditation scores extracted.
Case-selection boundary	Purposefully selected as the more formalized of the two eligible sites.	Purposefully selected as the more relationship-dependent of the two eligible sites.	Analytically informative prefecture-level hospitals outside megacities; not statistically representative.

CNAS, China National Accreditation Service for Conformity Assessment. Accreditation scope/status, staffing, workload, service descriptors, and shared context were checked against site records in January 2026. Rounded descriptors characterize context; site labels denote bundles rather than isolated interventions.

Hospital A held current China National Accreditation Service for Conformity Assessment accreditation in accordance with ISO 15189:2022 for a defined scope, with initial medical-laboratory accreditation granted in July 2024. The certificate, schedule of accredited activities, and current status were checked in January 2026; accreditation was not assumed to cover every activity discussed. Hospital B did not hold ISO 15189 accreditation during the study period. Both laboratories operated under Chinese national clinical-laboratory requirements, internal quality control, external quality assessment, competency authorization, and hospital quality-governance procedures. Accreditation was treated as a contextual characteristic rather than an intervention, exposure, or compliance outcome.

Sites were selected purposefully rather than as statistically representative hospitals. Pre-recruitment review identified comparable regional referral roles and clinical laboratory scope but contrasting dominant configurations. Hospital A had more formalized electronic acknowledgment, selected duplicate-order prompts, report-comment templates, documented escalation, and rule-review arrangements. Hospital B relied more heavily on direct access to laboratory leadership or section-level personnel and telephone-led coordination. These were sensitizing propositions rather than fixed site identities. Interviews actively tested where Hospital A depended on relationships and where Hospital B used formal rules. A standardized site-characteristics sheet was checked in January 2026 against administrative records, laboratory information system activity, staffing rosters, organizational charts, accreditation materials, and service records. Rounded scale descriptors were retained solely for context and were not converted into comparative rates.

### 2.5 Participants, workforce mapping, recruitment, and information power

Purposeful maximum-variation sampling sought participants with at least 1 year of employment at one site and direct experience of test ordering, prior-result review, specimen processes, result interpretation, critical or high-risk result communication, utilization management, laboratory consultation, quality governance, or laboratory information system and electronic medical record rule implementation. Candidate participants were identified through laboratory leadership, clinical liaisons, nursing supervisors, quality-management staff, information-system staff, and participant referral.

The two interviewers approached eligible candidates individually, using face-to-face contact, telephone calls, or private one-to-one WeChat messages. The initial contact introduced the research team, explained the purpose of the study, and offered a participant information sheet; subsequent scheduling followed the candidate’s preferred contact route. Invitations emphasized voluntary participation, confidentiality, and the value of critical or skeptical perspectives. A candidate who did not reply received one further invitation; no further invitation followed a refusal or non-response to that second contact. Managers and referrers facilitated identification but did not obtain consent or receive lists of those who accepted or declined. Contact messages were used for invitation and scheduling, not for collecting interview accounts or patient information. These recruitment channels were distinct from the face-to-face or secure online interview modes described in Section 2.6.

Because professional titles do not map directly across countries, the 16 laboratory participants were characterized by functional level, local position, qualifications, institutionally authorized duties, and decision authority. The group comprised 2 department leadership or director-level participants, 4 section or supervisory participants, 2 quality-management or authorized report-review participants, and 8 technical, bench, or preanalytical participants, distributed evenly across sites. One participant held a physician qualification and worked in clinical laboratory medicine; 15 were medical laboratory technologists, technicians, or preanalytical personnel. Educational preparation comprised 1 doctoral, 6 master’s, and 9 bachelor’s degrees; 4 held senior, 8 intermediate, and 4 junior Chinese professional titles. Degree and professional title did not independently determine authority. Report review, consultation, escalation, and policy duties were assigned through documented competency authorization. No laboratory participant had independent authority to diagnose disease or prescribe treatment. Table 4 maps these roles to internationally interpretable functional categories without asserting equivalence across jurisdictions.

**Table 4:** Functional mapping, qualifications, organizational level, and authority of laboratory participants (*n* = 16).

Functional category	Local positions included	Hospital A	Hospital B	Principal functions and authority	International interpretive note
Laboratory leadership/director level	Department director, deputy director, or equivalent	1	1	Department strategy, staffing, quality-system oversight, institutional representation, approval of selected policies, and high-level consultation; no independent treatment authority.	Functionally comparable to laboratory leadership, but not assumed to equal a pathologist, clinical scientist, or laboratory medical director elsewhere.
Section or supervisory level	Section lead, senior technologist, or physician-qualified laboratory participant with supervisory duties	2	2	Discipline-level workflow oversight, competency authorization, assay/specimen troubleshooting, consultation within documented competence, and escalation.	Maps to supervisory or section-level responsibility; interpretive authority varies across jurisdictions.
Quality-management or authorized report-review level	Quality manager, authorized report reviewer, or equivalent	1	1	Document control, internal/external quality monitoring, report verification, critical-result governance, audit preparation, and nonconformity follow-up.	Represents quality and authorization functions rather than a single globally standardized occupation.
Technical/bench or preanalytical level	Medical laboratory technologist, technician, specimen-reception, or preanalytical personnel	4	4	Specimen acceptance, testing, first-line validation, repeat/recollection triggers, and notification within defined authorization; escalation beyond scope.	Closest to bench-level laboratory personnel, but education, licensure, professional title, and autonomy differ across systems.
Total	Participants assigned to the primary function most relevant to the study	8	8	16 laboratory participants across four functional levels.	Categories support interpretation and confidentiality and are not asserted to be internationally identical.

Across the 16 laboratory participants, 1 held a physician qualification and worked in clinical laboratory medicine, and 15 were medical laboratory technologists, technicians, or preanalytical personnel. Educational preparation comprised 1 doctoral, 6 master’s, and 9 bachelor’s degrees; 4 held senior, 8 intermediate, and 4 junior Chinese professional titles. Qualifications are reported in aggregate to reduce deductive identification. Internal competency authorization, rather than degree or title alone, determined report-review, consultation, escalation, and policy duties. No laboratory participant had independent authority to diagnose or prescribe.

Sixty-two candidates were identified; 58 met eligibility criteria and were invited; 46 participated; 7 declined because of workload or lack of interest; and 5 did not respond after two invitations. No participant withdrew after interview. The final sample comprised 16 laboratory participants, 14 clinicians, 7 nurses or specimen-process coordinators, 5 quality or medical-administration staff, and 4 laboratory information system or electronic medical record staff. Hospital A contributed 24 participants and Hospital B 22. Professional experience ranged from 3 to 29 years, with a median of 12 years. Recruitment flow and stakeholder distribution are reported in Table 5.

**Table 5:** Recruitment flow and participant characteristics (*n* = 46).

Category	Hospital A	Hospital B	Total/details
Candidates identified	32	30	62 identified through maximum-variation and participant-referral routes.
Not eligible	2	2	4 did not meet predefined eligibility criteria.
Eligible and invited	30	28	58 invited directly by the research team.
Declined	4	3	7 declined because of workload or lack of interest.
No response	2	3	5 did not respond after two invitations.
Final participants	24	22	46; no withdrawal after interview.
Laboratory participants	8	8	16 across clinical chemistry, hematology/coagulation, immunology, microbiology, molecular diagnostics, preanalytics, report review, quality, and leadership; functional levels and qualifications are detailed in Table 4.
Clinicians	7	7	14 across emergency, intensive care, respiratory, infectious diseases, nephrology, endocrinology, obstetrics, pediatrics, and oncology.
Nurses/specimen-process coordinators	4	3	7 across ward nursing, emergency nursing, phlebotomy, specimen coordination, and critical-result relay.
Quality/medical-administration staff	3	2	5 across patient safety, quality management, clinical pathways, and medical affairs.
Laboratory information system/electronic medical record staff	2	2	4 across system maintenance, electronic ordering, alert logic, and data reporting.
Professional experience	Median 13.0 years; range 3–29	Median 11.5 years; range 4–27	Overall median 12.0 years; range 3–29.

Managers were not informed who accepted or declined. Leadership, liaison, and participant-referral routes may nevertheless have underrepresented staff outside those networks. Exact laboratory qualifications and titles were not attached to quotations.

Sampling adequacy was assessed through information power rather than data or code saturation (58). The research question was focused, participants had high domain knowledge, interviews were information-rich, and analysis deliberately compared roles, sites, functional levels, and discrepant accounts. After approximately 38 interviews, additional material mainly refined boundary conditions and site configurations rather than changing the analytic architecture. The final eight interviews were used to test negative cases, night and weekend vulnerabilities, override logic, the limits of cost-oriented metrics, and whether functional authority differed from nominal title. This stopping rationale concerns the explanatory adequacy and diversity of the material, not a claim that no further meanings could be generated or that all possible experiences had been exhausted.

### 2.6 Data collection

Long Yao (LY) and Zhuojun Deng (ZD) conducted the interviews; both had completed bachelor’s degrees in medical laboratory science. LY was female and ZD was male. Both were laboratory-trained researchers affiliated with the participating hospitals’ laboratory departments. LY had training in qualitative interviewing; ZD contributed familiarity with the hospital context from a laboratory perspective. In the wider team, Fuhua Luo (FL) was a master’s student affiliated with laboratory medicine, and Lei He (LH) was a master’s student in medical imaging. These descriptions distinguish completed qualifications from postgraduate enrollment and do not imply senior clinical or quality-management rank. The team’s analytic responsibilities are described in Section 2.8. Neither interviewer exercised direct supervisory authority over participants. Some participants knew one interviewer professionally; this relationship was disclosed during consent, recorded in the interview log, and considered in reflexive memos and cross-site interpretation.

Before consent and audio-recording, participants were informed of the interviewers’ names, institutional affiliations, laboratory backgrounds, and roles in the research. The interviewers explained that their academic interest was to understand how test requests, specimens, and results were interpreted, communicated, and acted on across professional roles, services, and shifts, and to identify feasible improvements in everyday coordination. Participants were told that the study sought accounts of both workable arrangements and difficulties, not evidence that more digitalization or a larger laboratory role was inherently better. It was not an assessment of individual performance, an accreditation audit, or a ranking of departments or hospitals. The interviewers clarified that they were acting as researchers rather than supervisors or hospital evaluators, welcomed accounts that challenged their assumptions, and explained that choosing not to participate or expressing criticism would not affect employment or training. This explanation made the research purpose and the interviewers’ interests explicit, separately from any pre-existing professional familiarity.

Thirty-six interviews were conducted face-to-face in private rooms and 10 through secure online meetings. No non-participants were present. Interviews lasted 42–96 min, with a median of 64 min. Four pilot interviews, involving one laboratory participant, clinician, nurse, and information-system participant, led only to wording refinements and were retained. The guide covered ordering and prior-result access, specimen integrity, interpretation, critical and high-risk results, communication routes, professional qualifications and functional authority, accreditation and quality-system context, digital-rule ownership, responsibility boundaries, night and weekend coverage, workload, boundaries with anatomical pathology, specific breakdown episodes, and feasible denominators and balancing measures for future evaluation. All interviews were audio-recorded with permission, transcribed verbatim in Chinese, and accompanied by field notes on context, non-verbal cues, role dynamics, and emerging analytic questions.

The guide moved from general workflow description to a recent concrete episode and then to counterfactual and measurement questions. Participants were asked who first recognized the problem, what artifact or person carried the information, who was expected to act, where responsibility stalled, what happened outside daytime hours, and what record could demonstrate closure without imposing disproportionate documentation. Later interviews incorporated emerging tensions, including click-as-closure, expertise concentrated in named individuals, generic comments, missing rule owners, and the possibility that cost-oriented metrics could reward underuse. This iterative design deepened negative-case testing without changing the core domains of the guide.

When participants described clinical events, names, exact dates, bed numbers, department identifiers, case identifiers, and unique timing sequences were removed. Quotations retain a broad stakeholder category and, where necessary, a broad functional band; site, specialty, exact title, seniority, and original transcript codes are suppressed. The labels E1-E9 identify the nine displayed source extracts, not individual participants, hospital membership, or transcript order. Reused extracts retain the same label and are explicitly identified as revisited evidence. Cross-site differences are reported in aggregate. Non-essential event details were altered only for identity protection and were not used to support mechanism claims.

### 2.7 Contextual document review

Thirty-six contextual workflow documents dated January 2023 to December 2025 were reviewed: 21 from Hospital A and 15 from Hospital B. Hospital A contributed four critical-result or specimen-process standard operating procedures, four report-comment or workflow templates, six digital-rule specifications or screenshots, two de-identified consultation-log extracts, and five quality-meeting or training records. Hospital B contributed three, three, two, three, and four documents in the same categories. The combined corpus comprised seven standard operating procedures, seven templates, eight digital-rule specifications or screenshots, five de-identified consultation-log extracts, and nine quality-meeting or training records. Site-characteristics sheets, organizational charts, staffing bands, and accreditation records were contextual verification materials and were not counted in the analytic corpus.

A structured evidence map linked each document to the workflow stage, responsible role, artifact, and claim it could support. Standard operating procedures established formal sequences; templates and screenshots showed how rules and comments appeared to users; consultation-log extracts indicated whether advice left a minimal trace; and quality or training records showed whether a problem entered review or learning. Documentary support was graded as confirming the existence of an arrangement, clarifying its wording, or demonstrating a documented review process. It was not used to infer actual frequency, adherence, or effectiveness.

Documents were not coded as an independent dataset, and no patient-level data, incident identifiers, departmental performance rates, or accreditation-compliance scores were extracted. They were used to map formal sequences, establish the existence and wording of rules or templates, and examine whether an account concerned an official arrangement, a local workaround, or a gap between policy and practice. When documents and interviews diverged, such as when a policy defined acknowledgment but participants described delayed clinical ownership, the divergence was retained as analytic evidence rather than resolved in favor of the document.

### 2.8 Reflexive thematic analysis, analytic lineage, and researcher roles

Analysis was conducted in Chinese using NVivo 14 for data management and retrieval. LY coded the full transcript set, maintained analytic memos, and prepared successive theme maps. ZD reviewed a purposively selected cross-site subset, tested site interpretations against discrepant cases, and checked contextual claims. FL maintained the document inventory, checked de-identification decisions, and mapped documentary evidence to site-level process claims. LH reviewed successive theme maps and contributed a medical-imaging perspective to discussions of diagnostic information, clinical authority, patient-safety language, and organizational accountability. Digital-public-health relevance and accreditation context were considered collaboratively. LH’s supervision and coordination concerned the research project, not participants’ clinical work or hospital quality management. Six scheduled analytic meetings addressed initial coding, role- and site-matrix review, negative cases, theme consolidation, model refinement, and final claim-status review. Coding differences were treated as prompts for interpretation rather than errors requiring an agreement coefficient.

An evolving coding record supported memoing and retrieval rather than inter-coder reliability. Event-near analytic labels included “report not enough,” “who owns the rule,” “nurse as bridge,” “one-click panel,” “alert without owner,” and “value counted only as speed.” These labels summarize meanings in the accounts; they are not all verbatim participant expressions. Comparison matrices retained stakeholder role, laboratory functional level, site, reported trigger, artifact, expected actor, responsibility gap, formal and informal route, and discrepant account. Their purpose was to preserve context and examine alternative interpretations, not to quantify theme prevalence or rank groups by agreement.

The analytic lineage connected three iterative interpretive moves. Event-centered coding first identified problems such as a technically valid result whose use remained uncertain, a call answered by someone unable to act, or a digital rule without continuing clinical ownership. Role- and site-comparison matrices then examined how those problems were understood and handled across contexts, preserving differences in meaning and authority. Boundary-infrastructure interpretation developed the four functions and their paired failure conditions. A subsequent, explicitly prospective design translation generated evaluation questions and candidate indicator families from these interpretations, participant suggestions about feasible records and balancing measures, and sociotechnical and laboratory quality-governance concepts. This last activity did not collect indicator data and was not an additional empirical dataset or a quantitative phase of thematic analysis. Table 6 links the displayed extracts and reported episodes to the interpretive moves and keeps future testing distinct.

**Table 6:** Evidence-to-interpretation lineage: displayed extracts, reported episodes, functions, and prospective questions.

Function / synthesis	Displayed extracts and reported material	Cross-case interpretation / limiting account	Proposed failure condition	Explanatory step	Prospective question / boundary
Epistemic qualification	E1: unfinished report. E2: specimen-specific potassium recollection. Other accounts: borderline confirmation and comment burden.	A technically valid result may still require a bounded next step. Some clinicians preferred fewer comments; not every abnormality requires conversation.	Unqualified uncertainty: consequential evidentiary limits remain implicit.	From the gap between measurement and usable meaning to qualification of inference; not a demonstrated outcome benefit.	When does advice make uncertainty usable, and when is it noise? Future inquiry; no actionability rate measured.
Jurisdictional authorization	E3: laboratory responsibility for reliability and limits. E4: clinician boundary between advice and disease assignment.	Bounded advice was valued; diagnostic overreach was resisted. Authority depended on recognizable competence, not degree or seniority alone.	Ambiguous or overstepped jurisdiction: advice is silenced, resisted, or misread as diagnosis.	From role-specific knowledge claims to authorization that enables collaboration without transferring clinical ownership.	Are advisory and decision roles recognizable in practice? Future assessment; no role-compliance rate measured.
Accountable handoff	E5: calling versus closure. E6: audit record versus responsibility. E7: result awaiting an owner. Paraphrased rejection/add-on and direct-call accounts.	Receipt, authority, and subsequent action were distinguishable. A workable direct-call account limited any claim of electronic necessity.	Unowned notification: information reaches an intermediary without owned action or escalation.	From relay and audit gaps to an explicit responsibility route; reported events were not linked to patient-level audits.	Does acknowledgment connect to an authorized next step across shifts? Future event tracing; no measured handoff effect.
Recursive institutionalization and learning	E8: ordering friction. E9: rule without continuing ownership. E6 revisited for dashboard interpretation.	Usable context and exceptions mattered. A sponsor-departure account showed how software could remain active without clear clinical ownership.	Orphaned artifact: technical persistence without responsibility for explanation, review, or change.	From ordering and maintenance accounts to continuing clinical ownership and a learning cycle.	Who maintains rationale, exceptions, and review decisions? Future assessment, not an accreditation score.
Coverage and burden	E7 revisited: nurse-mediated relay. E8 revisited: differential ordering effort. Other accounts: alert response, documentation, and concentrated consultation work.	A completed digital step can coexist with residual work absorbed by another role. Perceived friction is not a measured click count.	Hidden gaps in competent response or compensatory work despite apparent deployment.	Cross-function interpretation of access, authority, and who absorbs unfinished work; no workload measurement.	Who obtains support and who performs residual work? Future sampling by service, shift, and role.
Configuration coupling	E1 and E9 revisited together; aggregate site accounts and exceptions described in Sections 3.2–3.6.	Formal routes and contextual expertise had complementary perceived affordances. Staffing, accreditation, leadership, and digital history co-varied.	Traceable processes without usable expertise, or responsive expertise without durable responsibility and memory.	Abductive comparison of contextual bundles; quotation reuse adds an interpretation, not independent corroboration.	Do formal routes reach competent expertise and feed experience into review? Proposition for testing, not a verified necessary condition.

E1–E9 identify nine displayed source extracts, not nine necessarily distinct individuals or additional interviews. The same identifier is retained when an extract is revisited. Codes are analytic labels rather than presumed verbatim speech. Paraphrased episodes are not assigned to speakers of nearby quotations. Contextual documents establish formal arrangements, not event-level outcomes. The display separates situated material, interpretation, and future evaluation; it is not a frequency matrix or an independent dataset.

A claim-status review challenged each developing interpretation by asking whether it illuminated a reported breakdown, remained meaningful across relevant roles and sites, and accommodated an opposing or limiting account. These questions were prompts for reflexive scrutiny, not numerical thresholds for accepting themes. Cross-role recurrence was not treated as independent confirmation of a single recovered truth; differences in authority, access, and institutional loyalty remained relevant. Site-specific affordances were retained as configuration findings rather than promoted to universal mechanisms. In the separate evaluation agenda, candidates required a plausible criterion, future ascertainment frame, data source, responsible owner, and balancing risk. Those are requirements for later protocol development, not observed values or evidence that a theme is valid.

Discrepant cases were sought within every developing function. They included clinicians who preferred minimal laboratory involvement, laboratory participants who feared assuming diagnostic responsibility without full clinical context, nurses who described receiving critical information without decision authority, information-system staff who saw rules become unmaintained software artifacts, quality staff who distinguished an auditable call from owned action, managers who initially equated success with fewer orders, and participants who reported formal rules at Hospital B or relationship-dependent work at Hospital A. These cases specified when stewardship was trusted, resisted, or likely to fail and prevented the sites from being treated as a simple binary.

Explanatory strength was assessed through fit to the reported sequence, attention to contextual constraints, and the ability to account for exceptions. For example, a successful direct call limits an explanation that treats electronic acknowledgment as intrinsically necessary, while an acknowledged result without owned action limits an explanation that equates documentation with completion. Such contrasts support a function-centered interpretation of the communication problem, but cannot establish how often a configuration fails or whether one would outperform another after implementation. Because the material contains accounts of practice rather than direct observation or linked event audits, the proposed mechanisms remain revisable explanations, not verified causal pathways.

### 2.9 Translation, reflexivity, and trustworthiness

Quotations were selected after theme development to illustrate patterned meanings, event detail, role contrasts, and limiting accounts. Coding and theme construction remained in Chinese. A bilingual researcher compared English translations with source segments, and the team discussed ambiguous clinical and laboratory terms. Translation prioritized conceptual equivalence and retained conditional or skeptical language; minor grammatical editing did not change substantive meaning. The English quotations are translated direct speech, not claims that interviews were conducted in English. Each displayed extract has a role label and an E identifier; Table 6 links these extracts to the analysis. Section 3.6 revisits identified extracts to develop a cross-cutting synthesis, without treating reuse as additional testimony. Paraphrased episodes and cross-case explanations are presented as such, not placed in quotation marks or attributed to the speaker of a nearby quotation. Quotation selection makes interpretation inspectable; it does not estimate prevalence.

No repeat interviews were conducted and complete transcripts were not returned. A concise findings summary was instead discussed with eight participants drawn from laboratory leadership or supervision, technical laboratory work, clinical care, nursing, quality or medical administration, and information systems. This was treated as resonance testing rather than validation of a single correct interpretation. Feedback sharpened the distinction among transmission, receipt, acknowledgment, accountable action, and measured outcome and confirmed the need to explain local titles through function and authorization.

The team’s laboratory backgrounds could make analytical reliability and the visibility of laboratory work particularly salient, potentially privileging laboratory accounts over clinical, nursing, or information-system perspectives. LY and ZD’s site affiliations and professional familiarity could normalize local practice, leave apparently shared knowledge unexplained, or encourage institutional loyalty. FL’s document-focused contribution could privilege formal records over lived workflow. LH’s medical-imaging training offered a cross-disciplinary perspective on diagnostic information, but was treated as a situated contribution rather than superior interpretive authority. Differences between the researchers’ training positions and participants’ occupational authority could also influence what was explained, assumed, or withheld. Reflexive memos documented assumptions about laboratory value, clinical authority, accreditation, digital control, communication burden, cost reduction, and the tendency to treat electronic traceability as success. Interpretations were challenged by asking whether they appeared across roles and authority levels, whether an opposing case existed, whether an account might reflect institutional loyalty, and whether an arrangement remained usable at night, during turnover, or under workload pressure. Credibility, reflexive transparency, and the basis for assessing transferability were supported through maximum variation, two-site comparison, role matrices, discrepant-case analysis, document-supported process mapping, reflexive memoing, resonance testing, and a claim-status audit trail (59, 60). The audit trail makes interpretive decisions open to scrutiny; it does not imply that another team must reproduce identical themes or that documentary alignment is sufficient to establish causality.

### 2.10 Ethical considerations

Ethical approval was granted by the Ethics Committee of The First Affiliated Hospital of Shaoyang University (approval no. 20250407001), with site authorization from the Research Ethics Office of The Second Affiliated Hospital of Shaoyang University (authorization no. 20250409001). All participants provided written informed consent. Participation was voluntary, withdrawal was permitted before transcript anonymization, and audio files and transcripts were encrypted and accessible only to the research team. Because the hospitals are identifiable through author affiliations and several professional groups are small, quotations omit site, department, specialty, exact title, educational level, seniority, original transcript code, and unique event sequences. Displayed E identifiers label excerpts only and do not expose the underlying identity key.

## 3 Results

### 3.1 Participant profile and analytic architecture

Forty-six participants were included: 16 laboratory participants, 14 clinicians, 7 nurses or specimen-process coordinators, 5 quality or medical-administration staff, and 4 laboratory information system or electronic medical record staff. Hospital A contributed 24 participants and Hospital B 22; overall experience ranged from 3 to 29 years, with a median of 12 years (Table 5). Within the laboratory group, authority was layered rather than binary. Technical and preanalytical personnel acted within validated procedures and initiated escalation; supervisory, quality, and authorized-review personnel provided broader analytical or interpretive advice within documented competence; leadership approved policies and represented the service; and clinicians retained diagnosis, treatment, and final clinical-action authority (Table 4).

The analysis developed four infrastructure functions: epistemic qualification, jurisdictional authorization, accountable handoff, and recursive institutionalization and learning. Section 3.6 integrates evidence across these functions to examine coverage, burden, and configuration coupling. It is a cross-cutting interpretation, not a fifth independently established function. Each substantive subsection links translated direct speech to reported situations, contrasts, and a bounded explanatory claim. Table 6 records the links using excerpt identifiers E1-E9. Counts characterize the sample and context only; no theme-frequency rankings or process-performance estimates were produced.

### 3.2 Epistemic qualification: making uncertainty actionable

Participants distinguished an analytically valid output from information on which a receiving team could act. This distinction mattered when a request lacked a clear indication, collection conditions compromised a specimen, or an unexpected result conflicted with other information. The desired laboratory contribution was usually a bounded next step, such as recollection, confirmation, cautious interpretation, obtaining missing context, or escalation, rather than a complete diagnosis.

A supervisory or authorized-review laboratory participant explained: “A report can be analytically correct and still unfinished. If the number does not fit the specimen, the time point, or the patient, the report needs a conversation before it becomes a decision” (laboratory participant, E1). The account locates uncertainty between measurement and use: the assay can be complete while the clinical meaning of its output remains unsettled. Clinicians similarly valued comments naming a specific limitation and next step, while describing generic phrases such as “correlate clinically” as returning the interpretive burden without specifying what required correlation. Qualification therefore concerned the warrant for acting on a result, not simply adding more text to a report.

A clinician’s account of a night-shift potassium episode made this distinction concrete. The value was high, the draw had been difficult, and the specimen problem had to be reconciled with the possible treatment response: “The laboratory did not just say hemolysis; they asked about the draw and told us not to treat from that tube. The sample was recollected before medication was given” (clinician, E2). In this reported sequence, the laboratory contribution was a specimen-specific limit on inference and a recollection request, not a disease diagnosis. The account illustrates how collection information shaped the receiving team’s next step. It does not independently establish that withholding medication was appropriate in every comparable situation or that an adverse event was prevented; clinical urgency and patient outcomes were not audited.

A different, paraphrased account concerned a borderline infectious-disease result. The numeric value and reference interval were visible, but the confirmation route and timing conditions were not consistently apparent to the receiving service. A laboratory participant described calls after clinicians had already explained the preliminary result to patients. This account extends the distinction illustrated by E1: a technically released result can convey more certainty than the testing sequence warrants. Participants proposed context-sensitive confirmation guidance, while warning that a universal comment could become noise in low-risk or resolved cases. The borderline-result account and E1 are separate pieces of material, not a single reconstructed encounter.

Limiting accounts sharpened the interpretation. Some clinicians wanted fewer, more consequential comments rather than a comment on every abnormality; laboratory participants resisted inferring a clinical diagnosis from incomplete context. These concerns qualify the scope of E1 and E2: not every released report needs a conversation, and consultation should not substitute for a competent clinician’s assessment. Epistemic qualification is the interpretive function by which a material limitation or confirmation requirement changes what can defensibly be inferred and identifies a proportionate next step. Its failure condition is unqualified uncertainty, where the receiving service treats a request, specimen, or result as self-explanatory despite consequential limits.

Cross-site accounts suggested different ways of accomplishing this function. Hospital A participants described selected comments, delta checks, prior-result displays, and specimen rules as supporting reproducibility, while also describing generic text and fatigue. Hospital B participants emphasized access to laboratory leadership or section-level expertise, with support dependent on person and shift. Both patterns had exceptions: Hospital A sometimes used trusted calls, and Hospital B sometimes used formal comments or specimen procedures. These contextual comparisons do not attribute any displayed quotation to a particular hospital or establish comparative effectiveness.

### 3.3 Jurisdictional authorization: enabling advice without transferring diagnosis

Laboratory participation required authorization as well as expertise. Technical and preanalytical personnel could identify specimen or analytical problems and initiate defined notifications. Authorized-review, quality, and supervisory personnel could verify reports, explain limitations, recommend repeat or confirmation within competence, and escalate complex patterns. Laboratory leadership could approve policies and participate in high-level consultation. Clinicians integrated these inputs with the broader clinical record and retained diagnosis, treatment, and final clinical-action authority. The central tension was not whether the laboratory should speak, but what type of claim each role could legitimately make and document.

A laboratory participant described complementary responsibilities: “The clinician owns the diagnosis and treatment. The laboratory owns whether the test information is reliable and where its limits are. If the result is being used beyond those limits, silence is also risky” (laboratory participant, E3). Advisory restraint did not mean silence about a consequential assay or specimen limitation. The speaker distinguished a claim about the reliability and permissible use of information from a claim about the patient’s diagnosis. This made the boundary enabling as well as restrictive: it identified a reason to intervene without assuming responsibility for the whole clinical decision.

A clinician expressed the receiving-side limit: “If the laboratory says the sample is not reliable or that a marker needs confirmation, that helps. If the laboratory assigns a disease from one number, that crosses the line. The boundary is the kind of claim being made” (clinician, E4). The contrast concerns the kind of knowledge offered, not simply the speaker’s status. Other event accounts showed that a bench-level participant could stop or escalate a compromised specimen under a validated procedure, whereas greater seniority without relevant competency authorization did not automatically make someone the appropriate report reviewer. Clinicians could also bypass technically knowledgeable staff when their advisory authority was not recognizable. E3 and E4 illustrate the professional distinction; the other accounts show how its organizational expression could vary.

A paraphrased consultation sequence concerned a discordant marker pattern: technical staff identified and escalated the pattern, an authorized reviewer checked analytical and specimen conditions and recommended confirmation, and the clinician integrated the result into the differential diagnosis and documented the plan. The analysis interprets these as coordinated, bounded contributions rather than a transfer of diagnosis to the laboratory. Accounts of resistance when advice lacked an evidentiary basis or identifiable authorizing role limited this interpretation. The consultation sequence is not presented as an independently observed case or as the event underlying E3 or E4.

Hospital A participants described receiver definitions, escalation policies, templates, and documented consultation routes as making role boundaries more explicit, while also reporting bureaucracy when a rule did not fit a case. Hospital B participants described trust in named leaders or supervisors as permitting rapid advice, while exposing the route to absence, turnover, nights, and weekends. These accounts support jurisdictional authorization as a function through which boundaries can enable collaboration when recommendation, decision, action, documentation, and escalation are distinguishable. It fails when advice is silenced, exceeds competence, is misread as diagnostic ownership, or disperses responsibility into an undefined collective.

### 3.4 Accountable handoff: moving responsibility with information

Participants distinguished transmission, receipt, acknowledgment, and accountable action after release of a critical or high-risk result. A laboratory participant described the gap: “Calling is easy; closing the loop is hard. The difficult part is knowing whether the person who answered can act, whether the responsible doctor understood, and whether the patient was actually managed” (laboratory participant, E5). A reachable recipient, a clinician with decision authority, and a completed clinical response were not interchangeable states. The account locates the difficulty after successful contact: information can arrive without specifying who now owns the next step.

This distinction concerned responsibility rather than an intrinsic preference for telephone or electronic communication. A telephone answer or an acknowledgment click could establish contact while leaving the receiver’s authority and the subsequent action unresolved. Conversely, either medium could support a useful handoff when it connected information, an appropriate receiver, a documented decision, and escalation. E5 expresses the laboratory’s uncertainty about those later stages; it does not itself establish what happened to the patient.

Night and weekend accounts revealed who bridged that uncertainty. Laboratory personnel often reached the nurse station first; the nurse then had to locate a physician who might be in a procedure or covering several wards. A nurse or specimen-process participant put the ownership gap this way: “The system says notified, but the result is still waiting for an owner” (nurse/specimen-process participant, E7). The additional calls were part of the handoff, even when the system’s record ended at initial notification. Receipt by an available intermediary did not give that intermediary the authority to make the clinical decision.

Accounts of specimen rejection and add-on testing extended the problem beyond critical values. A unit could receive a rejection notice without a named person responsible for recollection, or receive an add-on response after the specimen was no longer suitable without a recorded decision about alternatives. These were paraphrased accounts of different workflows, not additional quotations from E5. The relevant concern is captured by the already quoted phrase “whether the person who answered can act” (laboratory participant, E5, revisited). Repeated notification cannot by itself resolve a mismatch between the reachable receiver and the person authorized to complete the next step.

A limiting account concerned a laboratory participant who reached a known on-call clinician directly, obtained read-back, and recorded the decision in an available communication field. It showed how a non-electronic route could connect authority with a traceable decision, not that calls were generally superior. Separately, a quality participant cautioned: “The call record helps an audit, but it does not tell us who accepted responsibility for the next decision. A closed record is not necessarily a closed clinical loop” (quality participant, E6). This contrast separates the medium used from the work accomplished. The positive episode describes an apparently workable responsibility route; E6 warns against inferring clinical completion solely from a complete record. Neither supplies an audited patient outcome.

Hospital A participants described stronger electronic acknowledgment for selected critical values, but also procedural closure; Hospital B participants described rapid direct calls and personal escalation with less consistent documentation. Against these configurations, the accounts of authority (E5), intermediary relay (E7), and audit visibility (E6) support a six-element handoff specification: identify the issue; identify an authorized receiver; acknowledge or read back; reach the responsible decision-maker; document action, justified non-action, repeat testing, or escalation; and escalate when responsibility stalls. The failure condition is unowned notification. This is the researchers’ explanatory and prospective specification, not a six-stage sequence whose completeness or effects were measured.

### 3.5 Recursive institutionalization and learning: governing rules as clinical objects

Participants related ordering behavior to workload, order-set design, access to prior results, and the effort required to choose a narrower test. A clinician described this interaction: “It is not that doctors enjoy ordering more tests. The large panel is one click and the thoughtful choice is ten clicks. Under pressure, the system teaches people what to do” (clinician, E8). The contrast between one and ten clicks expresses perceived friction; clicks were not independently counted. The account suggests how a digital environment can favor an easy action under pressure, rather than explaining every unnecessary test as a deficit in clinical knowledge.

Related accounts described repeat chemistry orders that were easier to place than locating a recent result, and broad panels that were easier to select than individually justified tests. Participants nevertheless resisted indiscriminate blocking because a repeat could be clinically necessary. They favored displaying the previous result, interval, and rationale while preserving a documented override. Read together with E8, these accounts frame a rule as an intervention in clinical work whose rationale and exceptions matter, not merely as a technical restriction. Neither reduced test volume nor low override use would, alone, establish an improvement.

Information-system participants emphasized responsibility for keeping rules clinically meaningful after activation. Evidence, thresholds, use cases, exceptions, assay changes, overrides, and retirement all required continuing judgment. One participant stated: “Every rule needs an owner. Otherwise, after six months nobody remembers why it was built” (information-system participant, E9). Ownership here refers to someone able and authorized to explain and reconsider the rule, not simply the technician able to change a setting. Quality staff similarly distinguished a technically active rule from one whose rationale, usability, risks, and revision history could still be explained. The reference to 6 months belongs to the participant’s account; it is not a measured rule-review interval.

Hospital A accounts described a selected duplicate-testing rule displaying the previous result, interval, rationale, and an override field. Participants linked acceptance to that information and a usable exception route. Hospital B accounts expressed interest in similar support but described reliance on telephone reminders without a stable review cycle. A contrasting Hospital A episode concerned an older rule whose sponsor had changed roles: overrides remained visible, but staff could not determine whether its threshold reflected continuing clinical intent. These separately reported situations give the ownership problem expressed in E9 organizational specificity. They do not show that every repeated override was justified or every older rule obsolete.

Participants also questioned dashboards equating stewardship with fewer tests or faster acknowledgment. In this context, the quality participant’s distinction is pertinent: “A closed record is not necessarily a closed clinical loop” (quality participant, E6, revisited). A reduced repeat-test count could conceal missed necessary testing, and a short acknowledgment interval could coexist with unresolved action. Quality and information-system accounts therefore linked metrics to an owner, an interpretation rule, balancing information, and a review decision. E6 is reused to expose the limits of a record-based inference, not as new testimony or evidence that quantitative evaluation is inappropriate.

The resulting function is recursive institutionalization and learning: rules, comments, metrics, and workflows remain clinically owned and revisable across episodes. Its failure condition is an orphaned artifact whose continuing technical presence conceals the absence of responsibility for interpretation, review, and a retain-revise-retire decision. The interaction between ordering friction (E8) and continuing ownership (E9), together with the limiting rule episode, grounds this interpretation. Accreditation and quality-system maturity may have contributed to Hospital A’s arrangements, but the design cannot separate them from scale, staffing, leadership, digital history, or culture.

### 3.6 Coverage, burden, and configuration coupling

This cross-cutting synthesis revisits the preceding evidence to ask who can obtain competent support and who performs the work when a formal route does not complete the task. The nurse’s statement, “The system says notified, but the result is still waiting for an owner” (nurse/specimen-process participant, E7, revisited), is especially revealing. In the night and weekend accounts, the continuously reachable nurse had to locate a physician whose availability was uncertain. Digital notification marked one completed step while the remaining relay was absorbed by another role. Coverage is therefore interpreted as access to an appropriately authorized response, not simply availability of the notification system. The accounts do not quantify the distribution of that work or establish shift-specific patient outcomes.

A related redistribution occurred before a result existed. The clinician’s earlier account expresses the burden directly: “The large panel is one click and the thoughtful choice is ten clicks. Under pressure, the system teaches people what to do” (clinician, E8, revisited). The account identifies differential effort, not a measured usability score. Alongside descriptions of alert responses, override documentation, and consultation concentrated in particular experts, it directs attention to whose work is simplified and whose becomes more demanding. A workflow can appear efficient at the point of ordering or notification while moving interpretive, relay, or maintenance work elsewhere.

The relationship between formal arrangements and accessible expertise can be seen by juxtaposing two previously presented extracts. The laboratory participant’s observation that “If the number does not fit the specimen, the time point, or the patient, the report needs a conversation before it becomes a decision” (laboratory participant, E1, revisited) identifies a limit to standardized output. The information-system participant’s warning, “Every rule needs an owner. Otherwise, after six months nobody remembers why it was built” (information-system participant, E9, revisited), identifies a different limit: a rule can persist after responsibility for its rationale has become unclear. We interpret their conjunction as a need to connect contextual judgment with an arrangement that preserves responsibility over time. Neither statement establishes that personal conversation is always necessary or that every rule requires the same maintenance process.

Cross-site comparison located these tensions in different organizational configurations. Hospital A accounts emphasized traceability and reproducibility together with generic comments, alert burden, and click-based closure. Hospital B accounts emphasized responsive access to known experts together with absence, turnover, after-hours vulnerability, and inconsistent documentation. Both hospitals used formal rules and personal communication, and exceptions qualified each dominant pattern. These aggregate contextual accounts support comparison of how the functions were organized; the de-identified quotations illustrate meanings and problems rather than independently verifying which hospital performed better.

Configuration coupling is consequently an explanatory proposition: formal routes may preserve continuity and memory while accessible expertise makes uncertain information usable, provided the routes actually connect people with the authority and capacity to respond. E1, E7, E8, and E9 are deliberately revisited evidence, not new speakers or additional interviews. Their analytic value lies in linking interpretation, relay, effort, and maintenance across the four functions. A documented route without reachable expertise, or responsive advice without a durable responsibility record, can leave different parts of the work unresolved. Figure 1 integrates these relationships; neither the distribution of burden nor the effects of coupling were measured.

**Figure 1:**
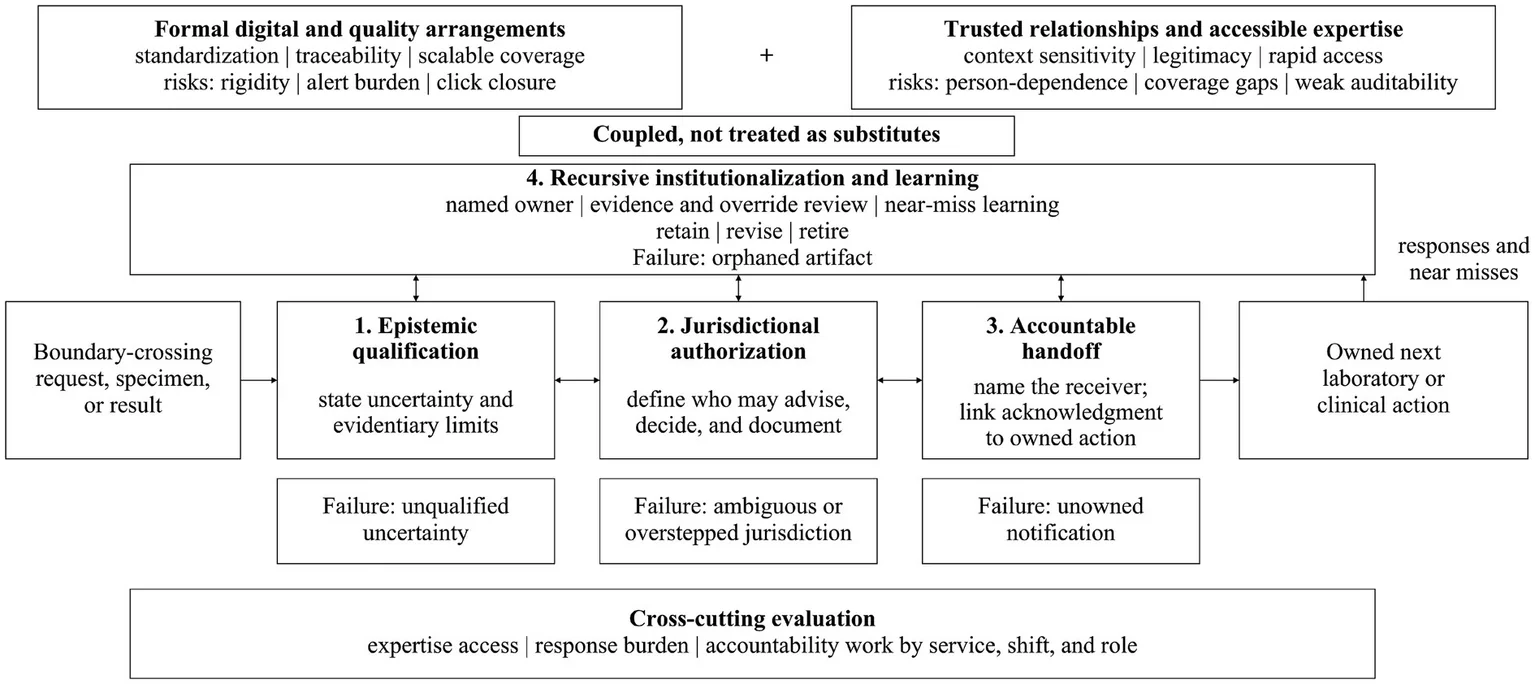
Boundary infrastructure connecting a laboratory request, specimen, or result requiring cross-boundary interpretation or action to an owned next laboratory or clinical action. The model distinguishes three analytically interdependent event-level functions within a recursive fourth function of institutionalization and learning. Bidirectional connectors mark analytical interdependence and do not imply an invariant temporal sequence. Formal digital and quality arrangements and trusted relationships are contextual configuration bundles with participant-perceived affordances and vulnerabilities; neither is treated as a causal exposure. Failure conditions identify where a boundary artifact ceases to carry accountable meaning. All connectors represent provisional explanatory relationships, not observed transitions, measured effects, or demonstrated causal mechanisms. Prospective qualitative and quantitative evaluation is required. The figure represents clinical laboratory medicine and excludes anatomical pathology.

## 4 Discussion

### 4.1 Principal findings

The study develops a critical realist explanation of why a clinical laboratory request, specimen, or result does not automatically become an accountable next action in digitally enabled regional referral hospitals. Four recurrent functions were developed through reflexive interpretation. Epistemic qualification made uncertainty and evidentiary limits actionable; jurisdictional authorization legitimized bounded advice while preserving clinical authority; accountable handoff moved responsibility with information; and recursive institutionalization maintained rules through ownership, monitoring, override review, and learning. Participants described uneven coverage and accountability work across services, shifts, and roles. Breakdowns were analytically productive because they revealed what each function had to accomplish.

The cross-site comparison generated a configuration proposition rather than a ranking. Participants associated Hospital A’s CNAS-accredited and more formalized configuration with traceability, reproducibility, and institutional memory, alongside generic comments, alert burden, and procedural closure. They associated Hospital B’s more relationship-dependent configuration with responsive, context-sensitive advice, alongside person-dependence, weaker after-hours coverage, concentrated expert workload, and limited auditability. Each configuration contained multiple co-varying conditions, and participant accounts exposed limitations in each. The resulting cross-case proposition is that standardized digital arrangements and accessible expertise perform different but complementary governance work.

### 4.2 Conceptual contribution: a diagnostic-stewardship account of boundary infrastructure

Boundary infrastructure is not proposed as a new term. Infrastructure scholarship explains durability, installed systems, and breakdown; boundary-work and invisible-work scholarship explain jurisdiction and hidden labor; and health-information infrastructure research shows that integration is an ongoing organizational achievement (28–35). The diagnostic-stewardship-specific contribution is a four-function account of what a boundary artifact must accomplish, the failure condition that exposes each function, and the evaluation questions and candidate measurement family that could examine it prospectively. An order prompt, specimen rule, report comment, call, acknowledgment record, or dashboard is not infrastructure by deployment alone. The model proposes that its infrastructural role depends on how it qualifies meaning, authorizes participation, transfers responsibility, and enters a learning cycle.

The model adds precision at three points. First, it distinguishes epistemic, jurisdictional, handoff, and recursive work rather than treating communication or digitalization as an undifferentiated enabler. Second, it pairs each function with a failure condition: unqualified uncertainty, ambiguous or overstepped jurisdiction, unowned notification, and an orphaned rule, metric, comment, or workflow. Third, it connects these failures to prospective indicator families without converting qualitative accounts into measured outcomes. The coverage-and-burden synthesis adds a further requirement: future evaluation should examine variation by service, shift, and role so that a deployed tool is not mistaken for equitable access or sustainable accountability work.

The explanatory claim is more specific than the proposition that good communication matters. At an interface, technical completion can be recognized before clinical responsibility is settled. If a system makes first receipt visible but leaves decision authority or escalation uncertain, another actor must compensate, or the next step can remain unowned. Similarly, a rule can continue to organize choices after responsibility for its clinical rationale has lapsed. These are conditional explanations inferred from the relay and rule-maintenance accounts, not demonstrated necessary or sufficient conditions. Their value is to specify where alternative explanations, such as workload, receiver availability, or poor targeting, should be examined rather than assuming that the presence of an artifact accomplishes the relevant function.

The model also clarifies the relationship between invisible work and value. Participants located much laboratory contribution outside the released report: clarifying a specimen, preventing an unsupported inference, locating a responsible clinician, explaining an alert, or revising a pathway after a near miss. Making this work visible does not mean counting every call. It means identifying the points at which expertise changes the usability, ownership, or learning value of diagnostic information and designing documentation that is proportionate to risk rather than burdensome by default.

### 4.3 Relationship to sociotechnical and implementation frameworks

The model is deliberately narrower than the Sittig-Singh sociotechnical model and NASSS. Those frameworks locate broad technological, human, organizational, and external determinants and explain implementation complexity, adoption, scale, spread, and sustainability (20, 21). The present model operates one level closer to the diagnostic event: it asks what functional work must occur as information crosses a boundary and where accountable meaning is lost. It can be nested within either framework rather than positioned as a substitute.

Analytic provenance is part of the contribution. Participant accounts supplied situated descriptions and event-near analytic codes; cross-role and cross-site matrices organized differences and recurrent problems; boundary-infrastructure interpretation developed higher-order function and failure labels; and a subsequent design step specified possible evaluation questions and indicators. Table 6 displays this chain. In critical realist terms, the explanatory ambition concerns conditions that might account for the reported problems, but the available accounts and contextual records do not establish those conditions as verified mechanisms (53, 54). The separation of reported experience, explanation, and prospective testing preserves the interpretive character of the findings while making the model open to empirical challenge.

### 4.4 Relationship to ISO 15189 and quality governance

The accreditation and quality-governance environment is an important contextual variable. Hospital A held CNAS accreditation in accordance with ISO 15189:2022 for a defined scope, whereas Hospital B did not; both were governed by national clinical-laboratory requirements and local quality systems. Accreditation may have contributed to Hospital A’s more explicit documentation, competency authorization, internal review, and rule maintenance. It cannot be separated from the hospital’s larger scale, staffing, information-system history, leadership, and organizational culture. The comparison therefore does not estimate an accreditation effect.

ISO 15189 and CNAS-CL02:2023 establish normative expectations for medical-laboratory quality and competence, including defined responsibility and authority, competent personnel, advisory activity, information management, risk management, evaluation, and continual improvement (36–38). The four-function model complements these requirements by explaining how they are enacted or frustrated at the clinical boundary. Jurisdictional authorization specifies how advisory competence can be used without transferring diagnosis or treatment authority; accountable handoff extends beyond result release to ownership of the next action; and recursive institutionalization links digital rules and metrics to override review, near-miss learning, and retain-revise-retire decisions. The model is not an ISO compliance scorecard.

### 4.5 Digital public health contribution

The public-health object of analysis is the institution-level governance of high-volume diagnostic information and scarce expertise within hospitals serving broad urban and county catchments. Relevant properties include diagnostic service capacity, coverage across departments and shifts, distribution of workload, comparability and accountability of information flows, and the ability to learn across large numbers of encounters (17–19). This focus is wider than the usability of a single hospital information-system module while remaining narrower than the performance of the complete regional referral network.

The accounts illustrate why digital maturity cannot be equated with accountable use. Hospital A had more formalized digital functionality, yet participants described generic comments, acknowledgment without action, alert fatigue, and burdens concentrated among staff required to respond or document overrides. Hospital B had less formal digital support, yet trusted consultation often made information usable; the same arrangement concentrated work on particular experts and weakened access outside daytime hours. The interpretation is that technology operated within existing governance configurations rather than independently securing legitimacy, ownership, consistent coverage, sustainable workload, or institutional learning.

The digital public health contribution is therefore an accountable-flow perspective. Digital systems should be assessed not only by adoption, uptime, or alert firing, but by whether evidentiary limits are visible, advisory authority is recognizable, responsibility reaches an actor who can act, service and shift coverage are protected, override remains clinically justified, and real-world experience feeds rule revision. These are institution-level capacities with population-serving consequences even when the empirical observation remains inside the hospital.

### 4.6 China-specific expressions and cross-system transferability

Several observations are tied to the Chinese setting: tertiary Grade-A classification and government hospital review; the local mix of physician-qualified laboratory personnel, medical laboratory technologists, technicians, section leads, and administrative titles; Chinese professional-title bands; hospital-based competency authorization; nurse-mediated critical-result relay; direct telephone access to known leaders or supervisors; and uneven ISO 15189 accreditation. These expressions should not be exported as universal role structures or preferred pathways.

The more transferable propositions are functional. The model proposes that uncertainty should be qualified sufficiently for a next step; advisory authority and its evidentiary limits must be recognizable; transmission and acknowledgment must be distinguished from accountable action; digital rules require named ownership and revision; and access to expertise and compensatory work must be examined by time, service, and role. A health system may allocate these functions to different professions or technologies, but their relevance, expression, and observable consequences require local assessment rather than assumption.

External validation should begin with functional-equivalence mapping. Each participating system should identify who may verify a result, advise on interpretation, decide diagnosis and treatment, acknowledge a high-risk result, own follow-up, maintain a digital rule, and review near misses. The four functions and failure conditions can then be assessed for conceptual relevance while indicators, thresholds, denominators, and responsible owners are adapted locally. Replication should include laboratories with different accreditation status, public and private governance, centralized and networked result management, and distinct professional jurisdictions. Anatomical pathology requires separate study because its specimen pathways, interpretive reporting, turnaround expectations, and diagnostic authority differ materially from the clinical laboratory workflows examined here.

### 4.7 Prospective validation and measurement

Table 7 is a prospective mixed-method evaluation agenda, not an empirical results table. Qualitative questions are placed alongside candidate process definitions to show what a future numerical change could and could not mean. No indicators, denominators, performance rates, theme frequencies, or hospital effects were calculated or validated. The presence of countable events does not itself determine a study’s epistemology: in a critical realist inquiry, measurement is one fallible representation of events, not an exhaustive account of the conditions producing them (53, 54). Here that measurement remains entirely prospective. The current study therefore has no quantitative strand and is not retrospectively reclassified as mixed methods.

**Table 7:** Prospective mixed-method evaluation agenda: qualitative challenges and candidate process definitions; no indicators measured in this study.

Future focus	Qualitative question / challenge	Candidate numerator / criterion	Future denominator / frame	Proposed records and ownership	Balancing risks
Appropriate repeat-order handling	When does cancellation reflect appropriate care rather than difficulty obtaining a necessary repeat? Examine workarounds and missed alerts.	Eligible repeat orders with clinically appropriate cancellation, completion, or justified override, assessed using prespecified review criteria.	All orders meeting electronic eligibility logic in the defined period, including eligible orders for which no alert fired.	Independent eligibility query linked to order, alert, prior-result, and override logs; clinical owner with laboratory/IT co-ownership.	Necessary repeats missed; delay; inappropriate cancellation; override burden; gaming; false triggers; service/shift differences.
Specimen integrity feedback	Does feedback clarify prevention and responsibility for recollection? Investigate falling rejection despite persistent specimen problems.	Rejected or recollected specimens with standardized cause coding and prevention guidance returned to the source unit.	All rejected or recollected specimens within a predefined target pathway and evaluation period.	Reception, recollection, and source-unit feedback records; preanalytics lead with nursing; assess coding consistency and patient matching.	Acceptance of poor-quality specimens; recollection delay; patient burden; feedback documentation without prevention.
Actionable interpretive consultation	How does advice shape decisions? Investigate more recorded consultations without better access or usable interpretation.	Eligible consultations linked to repeat, confirmation, reinterpretation, changed order, escalation, or a justified no-action decision.	Consultation episodes in a prospectively defined service-period sample; distinguish observed, recorded, and potentially missed episodes.	Prospective observation with minimal logs and sampled linked records; authorized laboratory reviewers and clinical liaison; report capture limits.	Response time; recording burden; generic comments; overreach; missed informal advice; unequal after-hours access.
Accountable critical-result handoff	Trace receiver authority and relay work. Examine prompt acknowledgment with unresolved action and failures despite an explicit route.	Target critical results linked to authorized receipt, responsible-clinician access, and documented action, justified non-action, or escalation within a local interval.	All target critical results released during the evaluation period, distinguished by urgency, service, and shift.	Linked laboratory/clinical timestamps, notes, acknowledgment, and escalation records; laboratory, nursing, clinical, and quality owners.	Mistaking acknowledgment for action; false urgency; interruption burden; inaccurate timestamps; night/weekend gaps.
Digital rule governance	Who can justify and revise the rule? Examine completed review forms without clinical reconsideration or an available owner.	Active rules reviewed when due, with an owner, override analysis, balancing review, and a documented retain-revise-retire decision.	All active stewardship rules due for review within the evaluation period.	Rule inventory, engine logs, minutes, and version history; clinical owner with laboratory/IT co-ownership; assess inventory completeness.	Alert fatigue; unsafe blocking; underuse; gaming; unequal effects; documented review without substantive learning.
Learning and visible value	Do completed changes resolve the problem or displace work? Seek eligible but excluded cases and persistently unresolved issues.	Eligible near-miss cases leading to a completed change in a rule, comment, route, training process, or responsibility assignment.	Cases meeting prespecified criteria within a defined ascertainment frame, including eligible cases not accepted for formal review.	Prospective screening linked to review, owner, and completed change; stewardship group; assess detection limits and time to change.	Cost-only interpretation; selective case capture; changes without effect; displaced work; underuse; unequal access.

All entries specify possible future evaluation. No numerator, denominator, rate, effect size, theme prevalence, compliance score, or hospital-performance comparison was calculated from these definitions. Local co-development must address clinical appropriateness, event ascertainment, missing capture, ownership, privacy, and burden. Qualitative inquiry tests the meaning and limits of numerical records. IT, information technology.

Future inquiry must distinguish testing an explanation from validating its chosen indicator. Prompt acknowledgment with unresolved ownership would challenge acknowledgment as a proxy for handoff; persistent breakdown despite verified receiver authority, access, and an explicit action route would also challenge the proposed explanation and invite investigation of additional conditions. More documented consultations without better access could reflect a recording change. Fewer duplicate orders accompanied by missed necessary tests would challenge an implementation’s claimed benefit. Interviews and observation should investigate these discordances rather than interpret numerical improvement as automatic confirmation of the present themes.

Definitions require co-development with the people who would collect and use the data. A denominator must identify a prospectively specified event population and ascertainment frame, not assume that a convenient log captures all eligible activity. Consultation evaluation needs particular care because unrecorded advice cannot be enumerated by the log whose incompleteness is under study. Prospectively sampled observation and linked records could assess capture; residual missingness should be reported rather than treated as zero. Criteria for action must recognize justified non-action and escalation, and ownership must be distinguishable from acknowledgment. Clinical appropriateness, identity and timestamp validity, privacy, usability, and recording burden require assessment before interpreting any candidate indicator.

A practical first use case should be bounded enough to connect digital events to accountable action: selected duplicate chemistry or coagulation orders, critical-result escalation, specimen recollection, confirmation after a borderline infectious-disease result, or defined interpretive comments. Depending on rollout constraints, a controlled before-and-after, interrupted time-series, or stepped-wedge design could compare implementation periods alongside qualitative process evaluation while preserving justified override. The protocol should predefine the clinical owner, intended users, inclusion logic, numerator, denominator, ascertainment method, escalation interval, review cycle, and balancing measures.

Repeat-order evaluation should identify electronically eligible orders independently of alert firing so that missed triggers remain visible, and should assess whether cancellation or completion was clinically appropriate. Consultation measurement should define the sampled service-period and distinguish observed, recorded, and potentially missed encounters. Near-miss evaluation should specify its ascertainment frame and eligibility criteria, retaining eligible cases not accepted for formal review. Critical-result analysis should distinguish urgency, service, and shift; acknowledgment alone cannot stand for subsequent action. These requirements concern future designs, not measurements available in the present dataset.

Balancing measures are essential because improvement in one visible metric can conceal harm elsewhere. Fewer repeat tests should be paired with necessary repeats and diagnostic delay; faster acknowledgment with time to documented decision or escalation; more comments with actionability and noise; lower rejection with inappropriate specimen acceptance; and rule performance with alert burden, underuse, gaming, and unequal effects. This design protects stewardship from being reduced to cost control or electronic compliance and keeps patient-facing consequences within the future evaluation pathway.

### 4.8 Governance implications for implementation

Implementation should begin by assigning ownership to a boundary-crossing use case rather than purchasing or activating a tool in isolation. A stewardship group should define the clinical problem, evidence base, intended users, advisory scope, responsible receiver, escalation interval, override conditions, and review cycle before specifying software behavior. Laboratory, clinical, nursing, quality, and information-system roles should be represented because each controls a different part of the pathway. The objective is not shared responsibility in the abstract, but explicit allocation of recommendation, decision, action, documentation, maintenance, and escalation.

Implementation also requires protected access to expertise. A formal trigger should route consequential uncertainty to a recognizable person or service with defined competence, including nights and weekends. Relationship-based consultation should be preserved where it adds context and legitimacy, but it should not depend on private knowledge of whom to call. A minimal consultation record can create institutional memory without converting every interaction into burdensome documentation. The appropriate record is the smallest one that identifies the question, bounded advice, action owner, and need for follow-up.

Finally, governance should treat overrides, near misses, and user burden as learning data. High override rates may indicate poor targeting, missing clinical context, or a changed evidence base; low override rates may reflect useful design or unsafe rigidity. Near misses should be screened with predefined criteria and linked to a completed change, not only discussed in meetings. Burden should be measured across roles because an apparently efficient digital process may shift work to nurses, night staff, or a small number of laboratory experts. These principles convert the four functions into a practical governance architecture while preserving the distinction between formative specification and demonstrated effectiveness.

### 4.9 Strengths and limitations

Strengths include maximum-variation sampling across five stakeholder groups, aggregate reporting of laboratory qualifications and functional authority, two purposefully contrasting sites, explicit recruitment flow, event-level accounts, active discrepant-case analysis, contextual process mapping, Chinese-language analysis, bilingual quotation checking, resonance testing, and an audit trail from reported event to explanatory function, failure condition, and prospective evaluation question. The item-level reporting maps distinguish described procedures, explicit non-use, justified methodological alternatives, and confidentiality-related adaptations. The study states its diagnostic scope, accreditation boundary, quality-governance environment, claim levels, and functional transferability logic explicitly.

Several limitations remain. Leadership, liaison, and participant-referral routes may have overrepresented staff interested in stewardship or comfortable criticizing digital processes; confidential invitations and discrepant-case sampling reduced but did not remove this risk. The sites differed simultaneously in scale, staffing, case mix, accreditation, quality-system maturity, information-system history, leadership, culture, and governance, so no observed difference can be assigned to one component. Both hospitals were located in the same prefecture and affiliated with the same university. Shared regulatory, professional, educational, referral, and labor-market influences may have increased contextual similarity and narrowed the range of institutional variation.

The study excluded anatomical pathology, county referring facilities, patients, public-health authorities, and external platform managers. Interviews and contextual documents did not provide direct workflow observation, linked event audits, patient outcomes, utilization rates, cost, delay, measured workload, or equity estimates. Documents established formal arrangements rather than independently verifying performance. Interviews could not show how rules and relationships changed longitudinally. Translation and protective generalization reduced organizational specificity. The nine displayed extracts make selected interpretations inspectable but do not represent nine necessarily distinct individuals; their reuse cannot establish independent corroboration or evidence distribution across hospitals. Broad role labels protect identities while limiting assessment of participant-level variation. Disclosure of researcher training, institutional affiliations, participant briefing, and recruitment routes makes potential influences on the accounts more visible, but cannot eliminate professional deference, self-selection, or socially desirable responses. Critical realism does not erase these limits or turn plausible organizational explanations into established causal mechanisms. Prospective, multi-site research is needed before inferring effectiveness or international generality.

## 5 Conclusion

In two Chinese regional referral hospitals, participant accounts described laboratory information that was technically available but not yet interpretable, appropriately authorized, or connected to an owned next action. Reflexive thematic analysis within a critical realist explanatory position developed four functions: qualifying uncertainty, authorizing bounded advice, transferring action ownership, and maintaining rules through continuing ownership and learning. The cross-cutting interpretation links these functions to access to expertise and the work of sustaining coordination across services, shifts, and roles. Formalized and relationship-dependent arrangements had different perceived affordances and vulnerabilities; the comparison supports a contextual explanation, not a hospital ranking or a component-specific causal effect. The empirical contribution consists of interpreted interview accounts and contextual process mapping. The separate indicators specify possible future tests. Diagnostic stewardship as boundary infrastructure is thus an inspectable, revisable account of how information becomes usable and responsibility becomes explicit, rather than a demonstrated intervention effect.

## Data Availability

Full interview transcripts are not publicly available because combinations of role, site, qualification, and event detail could enable deductive identification. De-identified analytic materials, including an evolving coding-record summary, functional role map, site-comparison matrix, document inventory, and indicator specifications, may be made available by the corresponding author upon reasonable request, subject to approval by the relevant ethics committees and participating institutions. Requests to access these materials should be directed to Lei He, 309999806@qq.com.

## References

[1] Zhi M Ding EL Theisen-Toupal J Whelan J Arnaout R. The landscape of inappropriate laboratory testing: a 15-year meta-analysis. PLoS One. (2013) 8:e78962. doi: 10.1371/journal.pone.0078962, 24260139 PMC3829815

[2] Bindraban RS ten Berg MJ Naaktgeboren CA Kramer MHH van Solinge WW Nanayakkara PWB. Reducing test utilization in hospital settings: a narrative review. Ann Lab Med. (2018) 38:402–12. doi: 10.3343/alm.2018.38.5.402, 29797809 PMC5973913

[3] Rubinstein M Hirsch R Bandyopadhyay K Madison B Taylor T Ranne A . Effectiveness of practices to support appropriate laboratory test utilization: a laboratory medicine best practices systematic review and meta-analysis. Am J Clin Pathol. (2018) 149:197–221. doi: 10.1093/ajcp/aqx147 PMC6016712 29471324

[4] Plebani M. Errors in clinical laboratories or errors in laboratory medicine? Clin Chem Lab Med. (2006) 44:750–9. doi: 10.1515/CCLM.2006.123, 16729864

[5] Laposata M Dighe A. Pre-pre and post-post analytical error: high-incidence patient safety hazards involving the clinical laboratory. Clin Chem Lab Med. (2007) 45:712–9. doi: 10.1515/CCLM.2007.173, 17579522

[6] Plebani M Lippi G. Improving diagnosis and reducing diagnostic errors: the next frontier of laboratory medicine. Clin Chem Lab Med. (2016) 54:1117–8. doi: 10.1515/cclm-2016-0217, 27101552

[7] Plebani M. The detection and prevention of errors in laboratory medicine. Ann Clin Biochem. (2010) 47:101–10. doi: 10.1258/acb.2009.009222, 19952034

[8] Fabre V Davis A Diekema DJ Granwehr B Hayden MK Lowe CF . Principles of diagnostic stewardship: a practical guide from the Society for Healthcare Epidemiology of America diagnostic stewardship task force. Infect Control Hosp Epidemiol. (2023) 44:178–85. doi: 10.1017/ice.2023.5, 36786646

[9] Morgan DJ Malani P Diekema DJ. Diagnostic stewardship: leveraging the laboratory to improve antimicrobial use. JAMA. (2017) 318:607–8. doi: 10.1001/jama.2017.8531, 28759678

[10] Hueth KD Prinzi AM Timbrook TT. Diagnostic stewardship as a team sport: interdisciplinary perspectives on improved implementation of interventions and effect measurement. Antibiotics (Basel). (2022) 11:250. doi: 10.3390/antibiotics11020250, 35203852 PMC8868553

[11] Zakhour J Haddad SF Kerbage A Wertheim H Tattevin P Voss A . Diagnostic stewardship in infectious diseases: a continuum of antimicrobial stewardship in the fight against antimicrobial resistance. Int J Antimicrob Agents. (2023) 62:106816. doi: 10.1016/j.ijantimicag.2023.106816, 37061101

[12] Baird G. The laboratory test utilization management toolbox. Biochem Med (Zagreb). (2014) 24:223–34. doi: 10.11613/BM.2014.025, 24969916 PMC4083574

[13] Baird GS Jackson BR Dickerson J Sikaris K Croal B Laposata M. What’s new in laboratory test utilization management? Clin Chem. (2018) 64:994–1000. doi: 10.1373/clinchem.2017.280214, 29632128

[14] Jackson BR. Managing laboratory test use: principles and tools. Clin Lab Med. (2007) 27:383–97. doi: 10.1016/j.cll.2007.03.009 17950895

[15] Plebani M Cadamuro J Vermeersch P Jovicic S Ozben T Trenti T . A vision to the future: value-based laboratory medicine. Clin Chem Lab Med. (2024) 62:2373–87. doi: 10.1515/cclm-2024-1022 39259894

[16] Epner PL Gans JE Graber ML. When diagnostic testing leads to harm: a new outcomes-based approach for laboratory medicine. BMJ Qual Saf. (2013) 22:ii6–ii10. doi: 10.1136/bmjqs-2012-001621, 23955467 PMC3786651

[17] Kostkova P. Grand challenges in digital health. Front Public Health. (2015) 3:134. doi: 10.3389/fpubh.2015.00134, 26000272 PMC4419711

[18] Iyamu I Xu AXT Gomez-Ramirez O Ablona A Chang HJ McKee G . Defining digital public health and the role of digitization, digitalization, and digital transformation: scoping review. JMIR Public Health Surveill. (2021) 7:e30399. doi: 10.2196/30399, 34842555 PMC8665390

[19] World Health Organization. Global Strategy on Digital Health 2020–2027. Geneva: World Health Organization (2025).

[20] Sittig DF Singh H. A new sociotechnical model for studying health information technology in complex adaptive healthcare systems. Qual Saf Health Care. (2010) 19:i68–74. doi: 10.1136/qshc.2010.042085, 20959322 PMC3120130

[21] Greenhalgh T Wherton J Papoutsi C Lynch J Hughes G A'Court C . Beyond adoption: a new framework for theorizing and evaluating nonadoption, abandonment, and challenges to the scale-up, spread, and sustainability of health and care technologies. J Med Internet Res. (2017) 19:e367. doi: 10.2196/jmir.8775, 29092808 PMC5688245

[22] Krasowski MD Chudzik D Dolezal A Steussy B Gailey MP Koch B . Promoting improved utilization of laboratory testing through changes in an electronic medical record: experience at an academic medical center. BMC Med Inform Decis Mak. (2015) 15:11. doi: 10.1186/s12911-015-0137-7, 25880934 PMC4344785

[23] Lacson R O'Connor SD Sahni VA Roy C Dalal AK Desai S . Impact of an electronic alert notification system embedded in radiologists' workflow on closed-loop communication of critical results: a time series analysis. BMJ Qual Saf. (2016) 25:518–24. doi: 10.1136/bmjqs-2015-004276, 26374896

[24] Li X Zou X Huang Y He Y. Clinical application of an intelligent outbound call system in the closed-loop management of critical values for inpatients. Am J Clin Pathol. (2026) 165:aqaf144. doi: 10.1093/ajcp/aqaf144, 41609691

[25] Tuijn CJ Msoka E Mushi DL Sumari-de Boer M Chilongola J van den Broek A. The interface between clinicians and laboratory staff: a field study in northern Tanzania. Afr J Lab Med. (2014) 3:127. doi: 10.4102/ajlm.v3i1.127 PMC5637763 29043178

[26] VanSpronsen AD Zychla L Villatoro V Yuan Y Turley E Ohinmaa A. Engaging laboratory staff in stewardship: barriers experienced by medical laboratory technologists in Canada. J Appl Lab Med. (2022) 7:480–94. doi: 10.1093/jalm/jfab103, 34599588

[27] Jalloh MB Vernooij E Street A. Invisible and undervalued: a qualitative study of laboratory workers' experiences and perceptions of laboratory strengthening in Sierra Leone. Afr J Lab Med. (2024) 13:a2292. doi: 10.4102/ajlm.v13i1.2292, 38840958 PMC11151379

[28] Star SL Strauss A. Layers of silence, arenas of voice: the ecology of visible and invisible work. Comput Support Coop Work. (1999) 8:9–30. doi: 10.1023/A:1008651105359

[29] Star SL Griesemer JR. Institutional ecology, translations and boundary objects: amateurs and professionals in Berkeley's Museum of Vertebrate Zoology, 1907-39. Soc Stud Sci. (1989) 19:387–420. doi: 10.1177/030631289019003001

[30] Star SL Ruhleder K. Steps toward an ecology of infrastructure: design and access for large information spaces. Inf Syst Res. (1996) 7:111–34. doi: 10.1287/isre.7.1.111, 19642375

[31] Bowker GC Star SL. Sorting Things out: Classification and its Consequences. Cambridge: MIT Press (1999).

[32] Star SL. This is not a boundary object: reflections on the origin of a concept. Sci Technol Hum Values. (2010) 35:601–17. doi: 10.1177/0162243910377624

[33] MacNaughton K Chreim S Bourgeault IL. Role construction and boundaries in interprofessional primary health care teams: a qualitative study. BMC Health Serv Res. (2013) 13:486. doi: 10.1186/1472-6963-13-486, 24267663 PMC4222600

[34] Lamont M Molnár V. The study of boundaries in the social sciences. Annu Rev Sociol. (2002) 28:167–95. doi: 10.1146/annurev.soc.28.110601.141107

[35] Ellingsen G Røed K. The role of integration in health-based information infrastructures. Comput Support Coop Work. (2010) 19:557–84. doi: 10.1007/s10606-010-9122-y

[36] International Organization for Standardization. ISO 15189:2022 Medical Laboratories—Requirements for Quality and Competence. Geneva: International Organization for Standardization (2022).

[37] China National Accreditation Service for Conformity Assessment. CNAS-CL02:2023 Accreditation Criteria for the Quality and Competence of Medical Laboratories (ISO 15189:2022, IDT). Beijing: CNAS (2023).

[38] National Health Commission of the People’s Republic of China. Measures for the Administration of Clinical Laboratories in Medical Institutions (issued 2006; amended by National Health Commission General Office Letter No. 560 [2020]). Beijing: National Health Commission (2020).

[39] Ministry of Health of the People’s Republic of China. Interim Measures for Hospital Accreditation, Ministry of Health Medical Administration Document No. 75 [2011]. Beijing: Ministry of Health (2011).

[40] National Health Commission of the People’s Republic of China. Standards for the Review of Tertiary Hospitals (2025 edition), National Health Commission Medical Administration Document No. 4 [2025]. Beijing: National Health Commission (2025).

[41] Graber ML Franklin N Gordon R. Diagnostic error in internal medicine. Arch Intern Med. (2005) 165:1493–9. doi: 10.1001/archinte.165.13.1493 16009864

[42] Schiff GD Hasan O Kim S Abrams R Cosby K Lambert BL . Diagnostic error in medicine: analysis of 583 physician-reported errors. Arch Intern Med. (2009) 169:1881–7. doi: 10.1001/archinternmed.2009.333, 19901140

[43] Newman-Toker DE Pronovost PJ. Diagnostic errors - the next frontier for patient safety. JAMA. (2009) 301:1060–2. doi: 10.1001/jama.2009.249, 19278949 PMC12396158

[44] Berwick DM Hackbarth AD. Eliminating waste in US health care. JAMA. (2012) 307:1513–6. doi: 10.1001/jama.2012.362, 22419800

[45] Brownlee S Chalkidou K Doust J Elshaug AG Glasziou P Heath I . Evidence for overuse of medical services around the world. Lancet. (2017) 390:156–68. doi: 10.1016/S0140-6736(16)32585-5, 28077234 PMC5708862

[46] Meng Q Mills A Wang L Han Q. What can we learn from China's health system reform? BMJ. (2019) 365:l2349. doi: 10.1136/bmj.l2349, 31217222 PMC6598719

[47] Yip W Fu H Chen AT Zhai T Jian W Xu R . 10 years of health-care reform in China: progress and gaps in universal health coverage. Lancet. (2019) 394:1192–204. doi: 10.1016/S0140-6736(19)32136-1, 31571602

[48] Greenhalgh T Robert G Macfarlane F Bate P Kyriakidou O. Diffusion of innovations in service organizations: systematic review and recommendations. Milbank Q. (2004) 82:581–629. doi: 10.1111/j.0887-378X.2004.00325.x, 15595944 PMC2690184

[49] Skivington K Matthews L Simpson SA Craig P Baird J Blazeby JM . A new framework for developing and evaluating complex interventions: update of Medical Research Council guidance. BMJ. (2021) 374:n2061. doi: 10.1136/bmj.n2061, 34593508 PMC8482308

[50] Braun V Clarke V. Using thematic analysis in psychology. Qual Res Psychol. (2006) 3:77–101. doi: 10.1191/1478088706qp063oa

[51] Braun V Clarke V. Reflecting on reflexive thematic analysis. Qual Res Sport Exerc Health. (2019) 11:589–97. doi: 10.1080/2159676X.2019.1628806

[52] Braun V Clarke V. One size fits all? What counts as quality practice in (reflexive) thematic analysis? Qual Res Psychol. (2021) 18:328–52. doi: 10.1080/14780887.2020.1769238

[53] Fletcher AJ. Applying critical realism in qualitative research: methodology meets method. Int J Soc Res Methodol. (2017) 20:181–94. doi: 10.1080/13645579.2016.1144401

[54] Wiltshire G Ronkainen N. A realist approach to thematic analysis: making sense of qualitative data through experiential, inferential and dispositional themes. J Crit Realism. (2021) 20:159–80. doi: 10.1080/14767430.2021.1894909

[55] Braun V Clarke V. Toward good practice in thematic analysis: avoiding common problems and be(com)ing a knowing researcher. Int J Transgend Health. (2023) 24:1–6. doi: 10.1080/26895269.2022.2129597, 36713144 PMC9879167

[56] O'Brien BC Harris IB Beckman TJ Reed DA Cook DA. Standards for reporting qualitative research: a synthesis of recommendations. Acad Med. (2014) 89:1245–51. doi: 10.1097/ACM.0000000000000388, 24979285

[57] Tong A Sainsbury P Craig J. Consolidated criteria for reporting qualitative research (COREQ): a 32-item checklist for interviews and focus groups. Int J Qual Health Care. (2007) 19:349–57. doi: 10.1093/intqhc/mzm042 17872937

[58] Malterud K Siersma VD Guassora AD. Sample size in qualitative interview studies: guided by information power. Qual Health Res. (2016) 26:1753–60. doi: 10.1177/1049732315617444, 26613970

[59] Tracy SJ. Qualitative quality: eight big-tent criteria for excellent qualitative research. Qual Inq. (2010) 16:837–51. doi: 10.1177/1077800410383121

[60] Mays N Pope C. Qualitative research in health care: assessing quality in qualitative research. BMJ. (2000) 320:50–2. doi: 10.1136/bmj.320.7226.50 PMC1117321 10617534

